# CD62L as target receptor for specific gene delivery into less differentiated human T lymphocytes

**DOI:** 10.3389/fimmu.2023.1183698

**Published:** 2023-08-14

**Authors:** Laura Kapitza, Naphang Ho, Thomas Kerzel, Annika M. Frank, Frederic B. Thalheimer, Arezoo Jamali, Thomas Schaser, Christian J. Buchholz, Jessica Hartmann

**Affiliations:** ^1^ Molecular Biotechnology and Gene Therapy, Paul-Ehrlich-Institut, Langen, Germany; ^2^ German Cancer Consortium (DKTK), Heidelberg, Germany; ^3^ Research & Development, Miltenyi Biotec B.V. & Co. KG, Bergisch Gladbach, Germany; ^4^ Frankfurt Cancer Institute, Goethe University, Frankfurt am Main, Germany

**Keywords:** L-selectin, receptor-targeted viral vectors, LV, chimeric antigen receptor, CAR T cells, ΔLNGFR, naïve T lymphocytes

## Abstract

Chimeric antigen receptor (CAR)-expressing T cells are a complex and heterogeneous gene therapy product with variable phenotype compositions. A higher proportion of less differentiated CAR T cells is usually associated with improved antitumoral function and persistence. We describe in this study a novel receptor-targeted lentiviral vector (LV) named 62L-LV that preferentially transduces less differentiated T cells marked by the L-selectin receptor CD62L, with transduction rates of up to 70% of CD4+ and 50% of CD8+ primary T cells. Remarkably, higher amounts of less differentiated T cells are transduced and preserved upon long-term cultivation using 62L-LV compared to VSV-LV. Interestingly, shed CD62L neither altered the binding of 62L-LV particles to T cells nor impacted their transduction. The incubation of 2 days of activated T lymphocytes with 62L-LV or VSV-LV for only 24 hours was sufficient to generate CAR T cells that controlled tumor growth in a leukemia tumor mouse model. The data proved that potent CAR T cells can be generated by short-term *ex vivo* exposure of primary cells to LVs. As a first vector type that preferentially transduces less differentiated T lymphocytes, 62L-LV has the potential to circumvent cumbersome selections of T cell subtypes and offers substantial shortening of the CAR T cell manufacturing process.

## Introduction

Genetic modification of T cells to express a chimeric antigen receptor (CAR) has emerged as an effective therapeutic treatment for patients with B cell hematological malignancies over the last few years. CAR T cells are generated from peripheral T cells isolated from the blood of patients. Based on the differential expression of CD62L, CCR7, CD45RA, and CD45RO, these peripheral T cells can be divided into five subsets: naive T (T_n_) cells, which are antigen-unexperienced; effector T (T_eff_) cells, which migrate to sites of inflammation and promote pathogen clearance; and memory T cells, which persist long-term to allow protection against subsequent infections. Memory T cells include stem cell memory (T_scm_), central memory (T_cm_), and effector memory (T_em_) cells (1). In humans, T cell differentiation follows a linear progression where less differentiated cells give rise to more differentiated progeny: T_n_ > T_scm_ > T_cm_ > T_em_ > T_eff_. During the differentiation of T_n_ toward T_eff_ cells, the proliferative potential and memory functions decline, while effector functions increase. Notably, the two markers, CD62L and CCR7, are only expressed on T_n_ and early-differentiated (T_scm_ and T_cm_) cells. During T cell isolation and subsequent cultivation, cells are usually activated using cytokines and stimulating antibodies to induce T cell proliferation and survival. In the past, IL-2 was most frequently used for cytokine support, thereby driving T cell cultures toward terminally differentiated T cells. More recently, IL-7 and IL-15 are applied to T cell cultures in an effort to maintain a more naïve- or memory-like T cell phenotype (2, 3).

Despite its promising results, CAR T cell therapy still needs to overcome various hurdles to become standard therapy for all patients in need. Automated processes have been developed to address the complicated manufacturing process (4). However, the most suitable T cell phenotype for CAR-mediated tumor therapy is a matter of debate. In general, naive and early-memory T cells are favored for cellular immunotherapy products due to their higher plasticity, longer persistence, and greater capability to proliferate and differentiate into highly cytolytic effector cells (5–8). Along this line, a beneficial antitumoral function and cell persistence were associated with a high amount of less differentiated CAR T cells not only in patients with B-cell malignancies but also in patients with neuroblastoma (3, 9–11).

For the generation of CAR T cell products, lentiviral vectors (LVs) pseudotyped with the glycoprotein of the vesicular stomatitis virus (VSV-G), harboring a broad tropism, are commonly used. Optimizing gene delivery through the engineering of vector particles offers the potential to improve and simplify the genetic modification of T cells. In this regard, receptor-targeted LVs (RT-LVs) specifically transducing CD3, CD4, or CD8 T cells have been described (12, 13). All three vector types were recently shown to mediate the generation of CAR T cells directly *in vivo* in humanized mouse models (13–16). RT-LVs use a cell surface protein of choice as an entry receptor, which can be achieved through pseudotyping with engineered glycoproteins from paramyxoviruses displaying a receptor-specific targeting domain, such as a single-chain antibody fragment (scFv) or designed ankyrin repeat molecule (DARPin) (17). However, the T cell-specific LVs available so far are not able to discriminate between the differentiation phenotype and exhaustion status of T cells.

Here, we describe the generation of an RT-LV that is specific for a T cell marker expressed on less differentiated T cells: CD62L. The specificity of this vector was mediated by displaying a CD62L-specific scFv on measles virus (MV)-based RT-LVs. The resulting CD62L-LV mediated efficient gene delivery and preserved a higher degree of less differentiated CAR T cells upon long-term culture. CAR T cells generated through short-term incubation with CD62L-LV controlled tumor burden in an *in vivo* setting.

## Results

A CD62L-specific scFv was derived from the antibody clone 145/15. Its sequence was fused to either the MV H protein or the NiV G protein via a (G_4_S)_3_ linker termed L3. Display on NiV G was performed with and without L3. All three constructs were equally well expressed at the surface of transfected HEK-293T cells (**Supplementary Figure 1**). For the production of CD62L-targeted LVs, HEK-293T producer cells were transfected with two envelope plasmids (one encoding MV H or NiV G fused to the targeting moiety and the other encoding the fusion protein MV F or NiV F), the lentiviral packaging plasmid, and the transfer vector encoding gfp. Small-scale stocks of vector particles harvested as unconcentrated supernatant were used for the transduction of target cells (HT1080_CD62L_ and HT1080_αHis_) and non-target cells (HT1080). Notably, HT1080_αHis_ cells are applicable target cells due to the presence of a His-tag at the C-terminal part of the CD62L-scFv fused to the NiV G and MV H protein.

**Figure 1:**
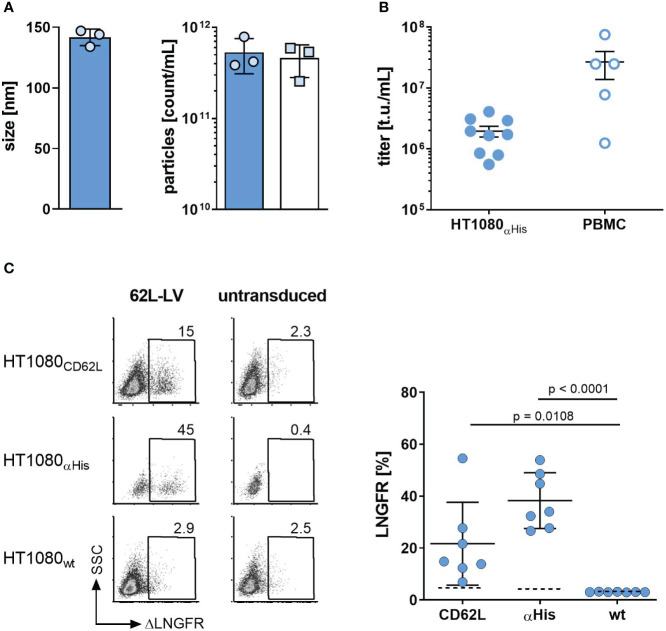
Basic characterization of 62L-LV. **(A)** Physical properties of 62L-LV vector stocks. Three independently produced 62L-LV stocks were analyzed for size (left panel) and particle concentration (right panel) by nanoparticle tracking analysis (filled bar; technical triplicates) or p24-ELISA (open bar, biological replicates). Means and standard deviations (SD) are depicted. **(B)** 62L-LV stocks were titrated on HT1080_αHis_ cells or activated human PBMC. Individual results of biological replicates and means with standard error (SEM) are plotted. **(C)** The indicated panel of HT1080 cells was incubated with 2.5 µL 62L-LV stock or left untransduced. Four days later, antibody staining against ΔLNGFR allowed for the detection of transduced cells by flow cytometry. Left panel: Representative dot plots for one vector stock. Right panel: Percentages of ΔLNGFR positive cells after transduction with seven different vector stocks. Dashed lines indicate detection levels for each individual cell line. Individual results as well as means with SD are plotted. Statistical testing was calculated by using ordinary 1-way ANOVA. WT = parental HT1080 cells.

While ^MV-L3^62L-LV was highly active in transducing both target cell types, both NiV glycoprotein-based LVs (^NiV-L3^62L-LV and ^NiV^62L-LV) were inefficient in gene delivery, especially on HT1080_CD62L_ cells (**Supplementary Figure 2**). This is potentially due to membrane distal binding of the scFv to CD62L as efficient gene delivery by NiV glycoprotein-based LVs requires membrane-proximal binding (18). Hence, ^MV-L3^62L-LV (hereafter termed 62L-LV) was chosen for further investigation. For all the following experiments, coding sequences for a second generation αCD19-CAR covering the 4-1BB costimulatory domain and the CD3ζ-signaling domain together with a truncated LNGFR (ΔLNGFR) reporter protein were packaged into LV particles. This vector was produced at a large scale, purified, and concentrated over a sucrose cushion. Vector stocks contained 2.6 – 7.9x10^11^ particles/mL, which were on average 142 ± 7 nm in size (**Figure 1A**). They were active in gene transfer as demonstrated by transduction of HT1080_αHis_ cells, on which an antibody recognizing the His tag on the particle envelope served as entry receptor (**Figure 1B**). CAR gene delivery was strictly dependent on CD62L expression since 62L-LV transduced HT1080_CD62L_ cells, which were genetically modified to overexpress CD62L but not the parental HT1080 cells, which did not express CD62L (**Figure 1C**; **Supplementary Figure 2A**).

**Figure 2:**
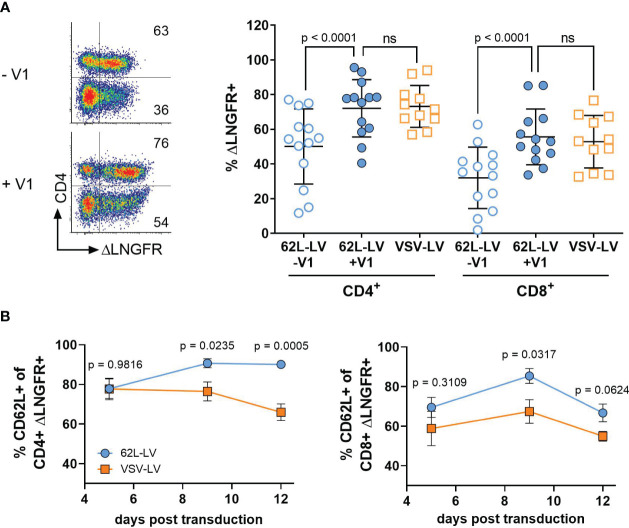
CAR gene delivery into primary lymphocytes by 62L-LV. Activated PBMCs were incubated with 62L-LV (blue dots) or VSV-LV (orange dots). **(A)** Transduction rates in the presence (+V1) or absence (-V1) of Vectofusin-1 as determined by ΔLNGFR expression. Left panel: Representative dot plots of 62L-LV transduced PBMC pre-gated for CD3^+^ cells. The percentage of ΔLNGFR expression presented as numbers in the individual gates refer to the CD4+ (upper gates) or CD4- cells (bottom gates), respectively. Right panel: results from seven different donors in four independent experiments analyzed 9 - 12 days post-transduction. For VSV-LV, V1 was not applied. Individual results of biological replicates and means with standard deviation (SD) are plotted. Statistical analysis was performed by using paired t-test for the comparison of 62L-LV +/-V1 and by using an unpaired t-test for the comparison of 62L-LV and VSV-LV. ns, not significant. **(B)** The total percentage of ΔLNGFR+ cells expressing CD62L is displayed for the CD4^+^ (left) and CD8^+^ (right) fractions. Transduction was performed in the absence of V1 for 62L-LV and VSV-LV, respectively. Cells from three different donors transduced with either vector in two individual experiments were tested for significant differences at each analysis time point individually by Fisher’s least significant difference (LSD) test. The mean with standard error (SEM) of eight biological replicates is plotted. The gating strategy is depicted in **Supplementary Figure 15**.

On primary human PBMC, gene transfer activity was higher than on HT1080_αHis_ cells (**Figure 1B**). Fractions of CD62L-positive T cells were donor-dependent and changed substantially during cultivation (**Supplementary Figure 3A**). All transduction experiments were performed 2 or 3 days after activation when CD62L levels ranged between 50-85% on T cells. Transduction of activated primary human PBMC obtained from various donors resulted in efficient gene transfer into CD4^+^ and CD8^+^ T lymphocytes (**Figure 2A**). Since CD62L can also be present on CD3-negative cells, especially B lymphocytes and monocytes (19, 20), we analyzed this cell fraction as well. At the day of transduction, up to 25% of cells were CD3-negative (**Supplementary Figure 3B**). This value decreased to below 2% upon cultivation (**Supplementary Figure 4A**). Gene transfer into these cells was detectable, however, at lower rates than on CD3-positive cells. Notably, VSV-LV was significantly more efficient in transducing these cells (**Supplementary Figures 4C, D**).

Gene transfer rates into primary T lymphocytes by 62L-LV were substantially enhanced through the addition of Vectofusin-1, resulting in more than 70% CD4^+^CAR^+^ T cells and 50% CD8^+^CAR^+^ T cells. Values were thus well comparable to those obtained with VSV-LV (**Figure 2A**). Higher numbers of transduced CD4^+^ over CD8^+^ T cells were also observed for non-targeted LVs pseudotyped with VSV-G or BaEV glycoproteins (21) and are, therefore, due to initially higher CD4^+^ T cell levels in PBMC cultures than in a particular property of 62L-LV (**Supplementary Figure 5**). In line with previous data on T lymphocytes, Vectofusin-1 was only beneficial for 62L-LV but not VSV-LV (**Supplementary Figure 4B**). CAR surface expression intensities were comparable for 62L-LV and VSV-LV, suggesting overall similar vector copy numbers in CAR T cells generated with both vector types (**Supplementary Figure 4E**). More importantly, even after cultivation of these cells for several days, CAR T cells generated with 62L-LV contained significantly higher numbers of less differentiated cells than CAR T cells generated with VSV-LV, as indicated by the higher percentage of CD62L^+^ cells (**Figure 2B**). This difference must be due to the targeting activity of 62L-LV since the levels of CD62L+ cells were identical in both T cell populations (**Supplementary Figure 6B**). Within the CAR+/CD4+ T cell fraction T_cm_ cells dominated, while similar levels of T_n_ and T_cm_ cells were present within the CAR+/CD8^+^ fractions for both vector groups (**Supplementary Figures 7A, B**). Compared to VSV-LV, fractions of T_cm_ cells were significantly higher for CAR T cells generated with 62L-LV 12 days post-transduction (**Supplementary Figure 7C**). The amounts of CAR-positive T cells slowly declined for both vector types within the CD8^+^ and CD4^+^ cell fractions over the cultivation period of 12 days, (**Supplementary Figure 6C**) possibly due to the absence of antigen stimulus and/or ΔLNGFR protein transfer contributing to the signals early after vector exposure (21).

**Figure 3:**
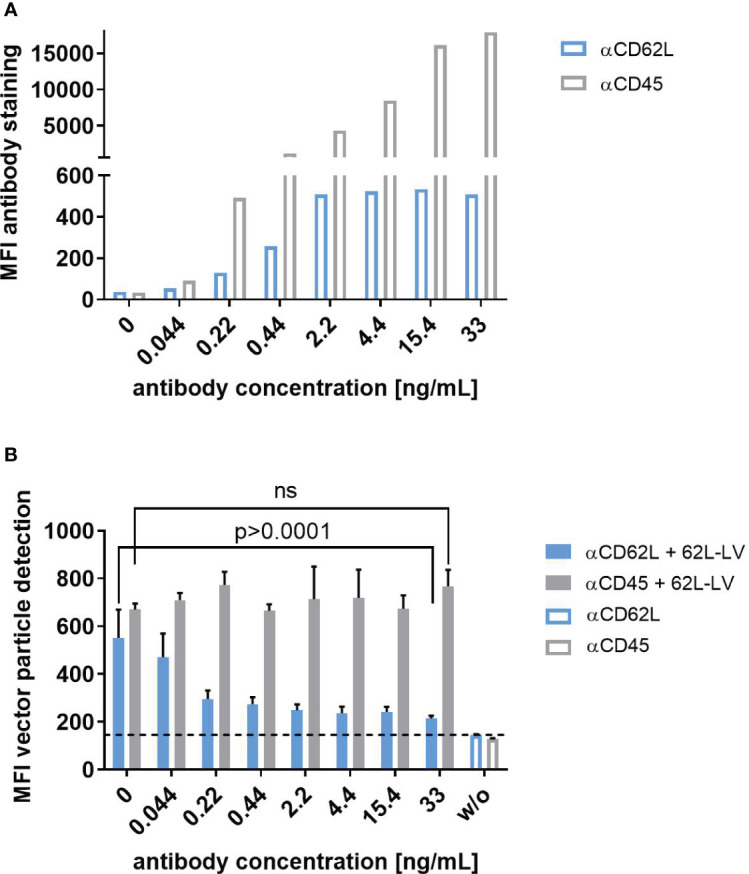
Selectivity of 62L-LV binding to primary T lymphocytes. Activated PBMC incubated either with the CD62L-specific antibody (blue bars) or with the CD45-specific antibody (grey bars) at the indicated concentrations before PBS (open bars) or 62L-LV vector particles (filled bars) were added for 30 min at 4°C. **(A)** Fluorophore-labeled αCD62L and αCD45 antibodies were used to determine the staining intensity of CD62L and CD45 on activated PBMC at the indicated concentrations by flow cytometry. Mean fluorescent intensities (MFI) are shown. N=1. **(B)** PBMC was pre-incubated with biotin-labeled antibodies before the addition of 62L-LV particles. After vector incubation, cells were stained with fluorophore-coupled αCD3 and αLNGFR antibodies to allow for the detection of vector-bound T cells by flow cytometry. Background MFI (dashed line) was determined from samples incubated with PBS (w/o) for all antibody concentrations. Means of MFI and standard deviations (SD) of three technical replicas are depicted. Statistical testing was performed by using 2-way ANOVA. ns, not significant.

To further assess the selectivity of 62L-LV on primary human PBMC, a blocking experiment with the parental CD62L antibody (145/15) or an unrelated antibody against CD45 was performed. Incubation of activated PBMC with increasing concentrations of either anti-CD62L or anti-CD45 resulted in a gradual increase in cell staining intensity for both antibodies (**Figure 3A**). CD62L staining peaked at a concentration of 2.2 ng/mL while anti-CD45 saturation was only about to be reached for the highest concentration applied although 100% of the cells were positive for CD45 also at lower antibody concentrations (**Figure 3A**; **Supplementary Figure 8**). The addition of 62L-LV vector particles to antibody pre-incubated cells showed that 62L-LV particle binding to cells decreased with increasing concentrations of anti-CD62L, while the unrelated antibody CD45 did not influence vector binding (**Figure 3B**). Notably, vector binding onto PBMC could be reduced close to background levels already at an anti-CD62L concentration of 2.2 ng/mL, demonstrating that 62L-LV binds specifically to CD62L on primary cells.

**Figure 4:**
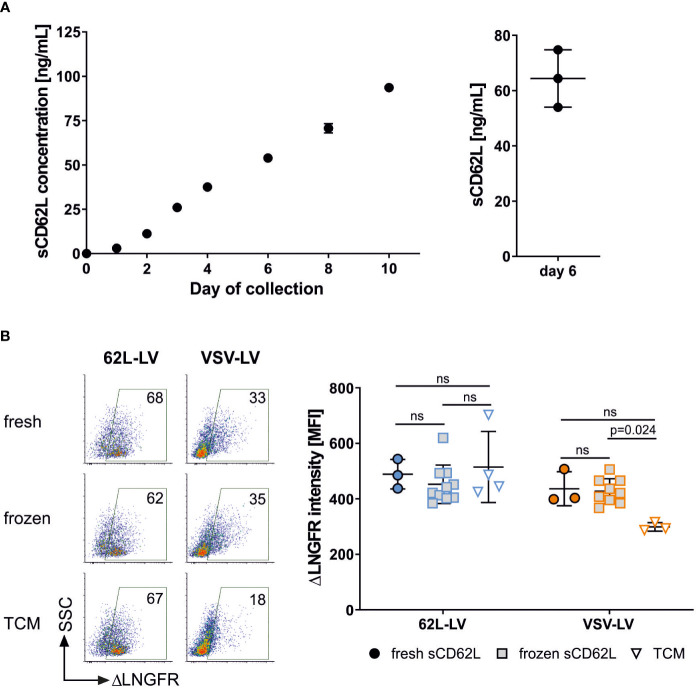
Shed CD62L does not influence vector binding. **(A)** Accumulation of sCD62L in the supernatant of PBMC. Frozen PBMCs were thawed, activated, and cultivated in the presence of IL-7 and IL-15. The complete supernatant of one well was collected on the indicated day and used for sCD62L quantification (left panel). The amounts of sCD62L present in three independent cultures on day 6 are shown in the right panel. Individual results and means and standard deviation (SD) are depicted. **(B)** 62L-LV (blue) or VSV-LV (orange) particles were incubated with fresh or frozen supernatant containing sCD62L (day 6 harvest) or cell medium (TCM) only. A mixture of vector stock and supernatant was incubated with activated PBMC for 30 min at 4°C. Flow cytometry was performed to analyze the content of vector-bound T cells by staining with fluorophore-coupled αCD3 and αLNGFR antibodies. Dot plots of vector-bound T cells are depicted in the right panel. The percentage of vector-bound cells is indicated. ΔLNGFR intensity [MFI] of vector-bound cells in three to 10 independent experiments is shown in the left panel. Individual results and means with SD are plotted. Statistical testing was performed by using 2-way ANOVA. ns, not significant.

During T cell activation and differentiation, CD62L is shed from the T cell surface. This has two consequences. First, CD62L levels in T cells strongly fluctuate in cell culture. It is therefore difficult to correlate the CAR gene and CD62L expression to prove the selectivity of 62L-LV after transduction of primary human PBMC. Second, shed CD62L (sCD62L) may bind to vector particles and reduce their gene transfer activity. Whether sCD62L hinders transduction by sequestering vector particles was subsequently analyzed in a binding experiment. As expected, accumulation of sCD62L in the supernatant of activated PBMC was observed for up to 10 days (**Figure 4A**). Supernatant from day 6, containing on average 64 ng/mL sCD62L, was used to pre-incubate 62L-LV particles prior to T cell binding. Interestingly, the pre-incubation of 62L-LV with either fresh or frozen supernatants containing sCD62L did not influence the binding of the vector particles to PBMC. Similar staining intensities of the reporter protein were detected regardless of whether vector particles were incubated with sCD62L-containing supernatants or fresh medium, indicating that sCD62L molecules present in cell culture supernatants did not alter the binding of 62L-LV to T cells (**Figure 4B**). Along this line, pre-incubation of vector and sCD62L did not impact the transduction efficiency of 62L-LV particles (**Supplementary Figure 9**). Transduction mediated by VSV-LV increased upon incubation with sCD62L containing supernatant (**Figure 4B**), which is an observation not further evaluated at this stage.

**Figure 5:**
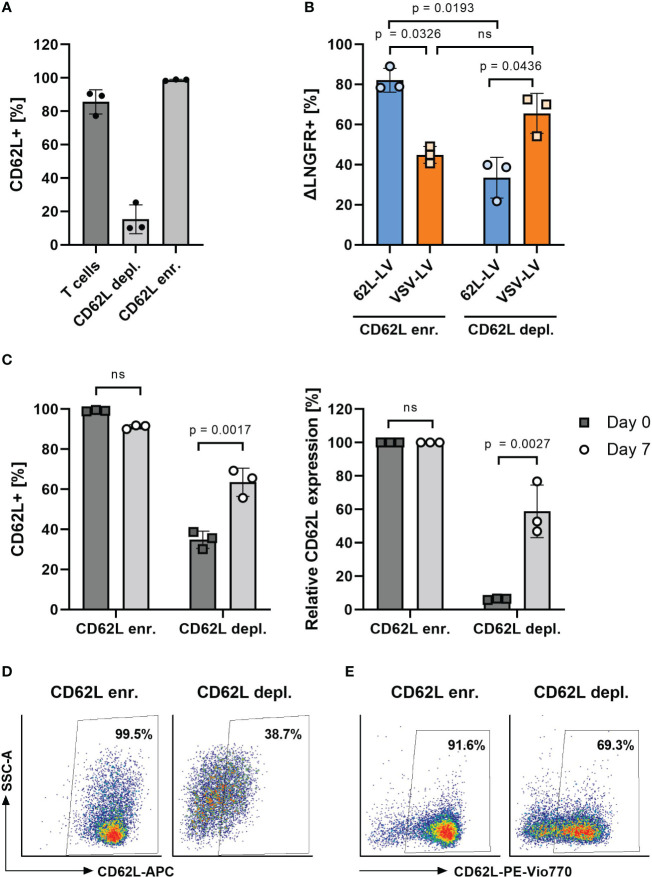
Transduction on CD62L-enriched and -depleted T cells. **(A)** CD62L frequency on activated T cells before (T cells) and after magnetic-activated cell separation using CD62L-APC (clone REAL163) and anti-APC microbeads into CD62L-enriched (CD62L enr.) and CD62L-depleted (CD62L depl.) fractions. **(B)** Percentages of ΔLNGFR+ cells 3 days after transduction of the separated cell fractions shown in panel A with 62L-LV or VSV-LV. **(C–E)** CD62L expression after separation. In an independent experiment, the percentage of CD62L+ cells was determined directly after separation (day 0) and upon 7 days of cultivation. Data are shown as measured **(C)**, left diagram), normalized to the values in the enriched fraction **(C)**, right diagram), and as exemplary FACS plots on day 0 **(D)** and day 7 **(E)**. Individual results as well as means with standard deviation are shown for three donors measured in technical triplicates. Statistical testing was performed by RM 2-way. ns, not significant.

Next, 3 days activated T cells were separated into CD62L-enriched and CD62L-depleted fractions and transduced with 62L-LV or VSV-LV. To prevent epitope masking by the CD62L antibody, the labeling antibody was enzymatically cleaved after cell separation. With this procedure, two fractions were obtained. The enriched fraction contained 98% and the depleted fraction contained 15% CD62L-positive T cells, respectively (**Figure 5A**). Upon cultivation, the fraction of CD62L-positive cells increased significantly in intensity and frequency in the depleted fraction, suggesting re-expression of CD62L on initially CD62L-negative cells (**Figures 5C–E**). Yet, 62L-LV resulted in significantly higher transduction on cells of the CD62L-enriched fraction than the depleted fraction (**Figure 5B**). Notably, there were approximately two-fold more transduced cells in the enriched fraction, while for VSV-LV, the result was the opposite (**Figure 5B**). There were also transduced cells in the depleted fraction (**Figure 5B**), which were most likely on-target transductions, either on the residual CD62L^+^ cells after separation (**Figure 5A**) or on cells re-expressing CD62L during exposure to vector particles.

After having provided evidence that 62L-LV specifically transfers CAR genes into CD62L-positive T cells, we tested the functionality of those CAR T cells. CAR T cells generated with 62L-LV exhibited a more naïve phenotype with significantly more T_n_ and T_scm_ cells than the cells transduced with VSV-LV 3 days post-transduction (**Supplementary Figure 10A**). In agreement with a higher content of T_eff_ and T_em_ CAR T cells, the killing of CD19^+^ tumor cells was more efficient with CAR T cells generated through VSV-LV (**Supplementary Figure 10B**). Yet, there was a significant killing detectable also for CAR T cells generated with 62L-LV even at a low ratio of effector to target cells (**Supplementary Figure 10B**). Notably, these CAR T cells contained a slightly higher level of CCR7-positive cells not only before but also after the killing assay (**Supplementary Figures 10C, D**).

**Figure 6:**
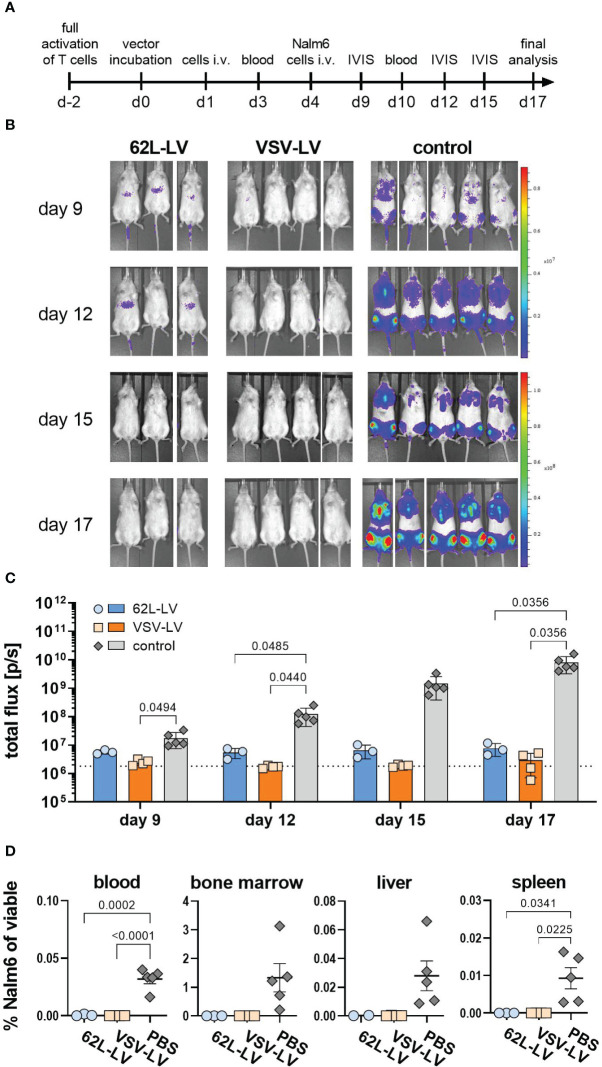
Antitumoral activity of CAR T cells generated with 62L-LV. **(A)** Experimental setting. PBMC were activated for two days prior to 24h incubation with 62L-LV, VSV-LV, or PBS (control) and injected i.v. into NSG mice (n=3 per group). Nalm6 cells were injected on day 4 of post-adoptive cell transfer and their growth was monitored by bioluminescence imaging (BLI). **(B)** Monitoring for tumor load by BLI at the indicated days after adoptive cell transfer. Ventral images of each mouse are depicted. **(C)** Total body flux quantified at the indicated time points for the 62L-LV group (blue), the VSV-LV group (orange), and the control (grey). Individual results and mean with standard error (SEM) are plotted. The dotted line represents the background signal of mice without imaging substrate. Ordinary two-way ANOVA was used to determine statistics. P-values are indicated when below 0.05. **(D)** Cells isolated from the blood and organs of each mouse were analyzed by flow cytometry for viable, CD45 negative, CD19, and EBFP double-positive Nalm6 cells. The percentage of Nalm6 positive cells of all viable cells is depicted. Individual results and means with standard error (SD) are plotted. Ordinary one-way ANOVA was used to determine statistics. P-values are indicated when below 0.05.

**Figure 7:**
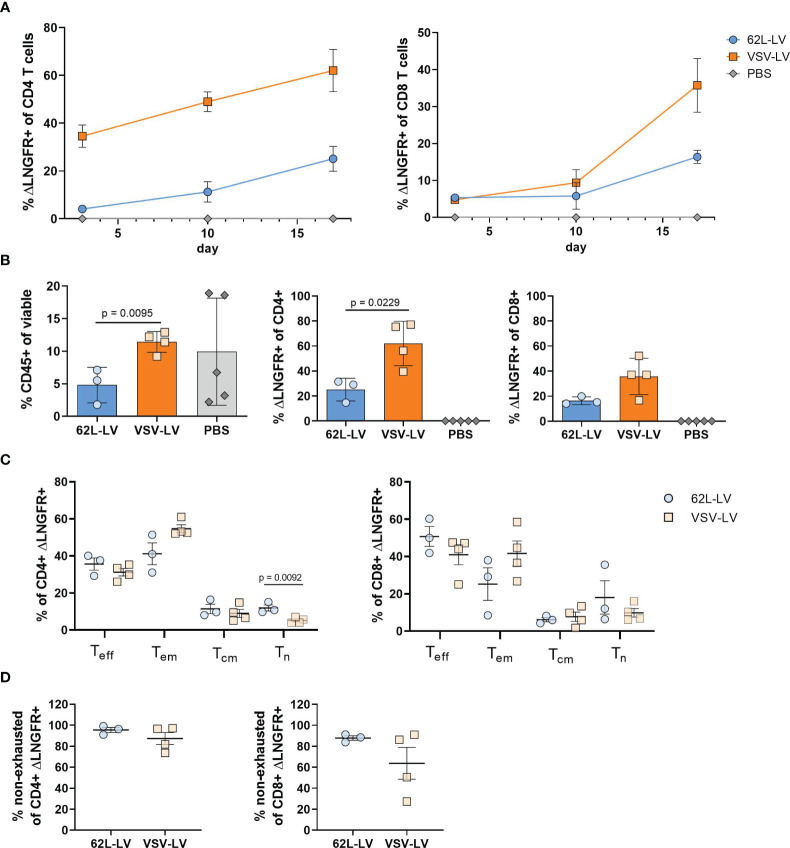
Characterization of CAR T cells from the *in vivo* experiment **(A)** Monitoring of CAR T cells in the blood. The fraction ΔLNGFR+ cells within human CD4^+^ or CD8^+^ T cells was determined by flow cytometry on days 3, 10, and 17 of the *in vivo* experiment. Gating on viable human CD45^+^, CD3^+^, and respective lineage marker-positive cells was performed. Only samples with at least 20 events in the CD4^+^ or CD8^+^ gates were considered. Mean values with standard error (SEM) are depicted. N=3. **(B-E)** Cellular composition in the blood at the final analysis. Frequencies of human CD45^+^ cells (left), ΔLNGFR+/human CD4^+^ (middle), and ΔLNGFR+/human CD8^+^ (right) are shown in **(B)**, those of Teff, Tem, Tcm, Tn within ΔLNGFR+/human CD4+ (left), and ΔLNGFR+/human CD8+ (right) in **(C)**, and frequencies of non-exhausted ΔLNGFR+/human CD4+ (left) and ΔLNGFR+/human CD8+ (right) as determined by double-negative TIM-3 and LAG-3 expression in **(D)**. Individual results of each mouse and mean values with standard deviation (SD) are depicted. Unpaired t-tests were performed to determine statistics. P-values are indicated when below 0.05.

In the next step, antitumor activity *in vivo* was investigated for CAR T cells generated with 62L-LV or VSV-LV. CAR T cells were short-term generated by 24h incubation of 2 days activated PBMC with equal volumes of 62L-LV and VSV-LV vector stocks and subsequently administered to NSG mice via tail vein injection. The vector doses applied reflected roughly identical particle numbers and an approximately seven-fold higher MOI for VSV-LV (**Supplementary Table 3**). Yet, a higher amount of CAR^+^ T cells was detectable in the 62L-LV group upon cultivation of the vector-cell mix for an additional two days (**Supplementary Figure 11C**). To demonstrate the functionality of the short-term generated CAR T cells, Nalm6 cells (luciferase-encoding CD19-positive target cells) were intravenously injected into the mice 3 days later, and tumor growth was monitored by bioluminescence imaging (BLI). A schematic timeline of the experimental set-up is presented in **Figure 6A**. Tumor growth was clearly constrained in both vector groups, while a steady increase of tumor mass, reflected by a more than 100-fold increase in luciferase signal, was observed in all control animals (**Figure 6B**). Quantification of signals revealed that tumor load in both vector groups was at or slightly above the background over all the days of analysis (**Figure 6C**). Notably, signals in animals that received VSV-LV-treated T cells were slightly reduced compared to those receiving 62L-LV-treated cells, but this difference was not significant. At day 17 of post-adoptive cell transfer, no tumor cells were detected in the blood, bone marrow, liver, and spleen of the sacrificed mice of both vector groups, while tumor cells were present in various organs of all control animals (**Figure 6D**). Along this line, proliferation of CAR T cells was observed in the blood over time of animals having received 62L-LV- or VSV-LV-incubated PBMC (**Figure 7A**; **Supplementary Figure 12**). Interestingly, higher proportions of CAR T cells and human CD45^+^ cells were observed in the blood (**Figure 7B**), spleen, bone marrow, and liver for the VSV-LV group at day 17 (**Supplementary Figure 13**). Interestingly, CAR T cells found in the periphery of mice in the 62L-LV group showed a tendency for a more beneficial cell composition regarding phenotype and cell exhaustion. Significantly, higher frequencies of T_n_ cells and fewer T_em_ cells were determined within the CD4+/LNGFR+ T cells in particular (**Figure 7C**). With respect to exhaustion, CD8+ CAR T cells derived from 62L-LV transduction exhibited a trend for lower levels of LAG-3 and TIM-3 (**Figure 7D**). In the spleen and bone marrow, the vast majority of CAR T cells showed the typical cytotoxic-associated phenotype, while less differentiated T cells were hardly detectable (**Supplementary Figures 14A, B**). Yet, CD8+ CAR T cells showed a tendency for reduced exhaustion when transduced with 62L-LV (**Supplementary Figures 14C, D**). In conclusion, in the applied animal model functional CAR T cells can be generated with 62L-LV by short-term *ex vivo* exposure to vector particles.

## Discussion

For any CAR T cell therapy, generating a product with high safety and efficacy in terms of longevity, engraftment, and antitumor-effector function is the ultimate goal. The design and cellular composition of the CAR T cell product are essential parameters defining these key therapeutic features. Parameters affecting CAR T cell function are, e.g., the choice of co-stimulation, the ratio between CD4^+^ and CD8^+^ CAR T cells, CAR T cell differentiation status, and the amount of exhausted CAR T cells (22, 23). This paper describes a novel gene transfer vector termed 62L-LV, which specifically transduces CD62L-positive cells, thus offering the potential to preferentially generate CD62L^+^ CAR T cells without the need for preselection of defined T cell subsets. Importantly, the newly generated 62L-LV vector could be robustly produced with regard to particle size, concentration, and functional titer. With an average size of 142 nm and 10^11^ particles/mL, the size and concentrations of 62L-LV stocks lay in the expected ranges of previously established RT-LVs (18, 21, 24–26). Functional titers of concentrated 62L-LV batches encoding the CD19-CAR were on average above 1x10^7^ t.u./mL on human PBMC; thus, they were approximately one log higher than on HT1080_αHis_ cells. This difference in gene transfer activity illustrates that titer determination depends on the particular experimental conditions including the cell type, used transgene, and transduction condition. Functional titers can therefore not be compared to those of other vector types. Gene transfer into primary human PBMC with 62L-LV was as efficient as with VSV-LV while resulting in a significantly higher proportion of less differentiated CAR T cells upon long-term cultivation.

As CD62L is a differentiation marker, its expression changes throughout the T cell lifetime and activation status. CD62L is regulated by transcriptional shutdown and shedding from the cell surface upon T-cell activation (27, 28). Therefore, direct proof for the selectivity of 62L-LV on primary cells is difficult since transduced CD62L^+^ cells might have turned CD62L negative when detecting gene expression. To address this issue, we pursued a variety of experimental strategies all supporting that 62L-LV is as selective for target-receptor positive cells as other RT-LVs, such as CD8-LV (29). We found that: i) T cells transduced with 62L-LV contained significantly higher proportions of CD62L^+^ CAR T cells than those generated with VSV-LV, ii) 62LV use CD62L as an entry receptor as demonstrated on engineered cell lines, iii) the transduction levels correlated positively with enrichment of CD62L^+^cell fractions from donor PBMC, and iv) vector particle binding to primary T lymphocytes was blocked by the parental CD62L-specific antibody from which the targeting domain of 62L-LV was derived.

An interesting finding of our study was that 62L-LV particles were not blocked by shed CD62L. This was unexpected, as it is known that the binding capacities of CD62L to target molecules are retained after cleavage (30). Various reasons might be causative for this finding. The concentration of sCD62L in cell culture supernatant was lower (50 ng/mL) than in the serum of healthy individuals (0.8 – 2.3 µg/mL) (30). In addition, sCD62L is known to aggregate (31), which further reduces the amounts of molecules available for binding of 62L-LV. Even more relevant, it has been suggested that the conformation of sCD62L differs from that of membrane-associated full-length CD62L since a monoclonal antibody directed against an epitope in the EGF-like domain of CD62L was able to bind to the cell surface-associated CD62L but not the soluble form (30). The same may hold true for the 145/15 antibody. As a consequence, 62L-LV would be specific for CD62L but not sCD62L. Regardless of the exact mechanism, we have proven that 62L-LV transduces T lymphocytes also in the presence of sCD62L.

CD62L is expressed on most circulating leukocytes, like B lymphocytes, neutrophils, monocytes, eosinophils, immature thymocytes, and a subset of NK cells as well as hematopoietic progenitor cells (19, 20, 32–35). This holds true also for certain malignant cells, e.g., B-ALL or NHL (36–38). Accordingly, all these cells are potential targets for 62L-LV if exposed to the vector. While binding of vector particles to these cells is highly likely, and may in the case of CD19-positive B cell lymphomas additionally be supported by vector particle-incorporated CD19-CAR (21, 39) binding is not sufficient for successful transduction. Binding via additionally incorporated non-fusogenic transmembrane proteins (such as the CD19-CAR here) is supposed to be rather inefficient in mediating membrane fusion and cell entry (40). Even after successful membrane fusion, post-entry blocks mediated by restriction factors can prevent transduction. An example is SAMHD1, which blocks early reverse transcription of LVs, especially in monocytes (41, 42). While further studies on 62L-LV-mediated transduction of myeloid cells and more importantly CD62L-positive tumor cells will be required, it appears well conceivable that the previously described LVs targeted to the T-cell markers CD8, CD4, or CD3 are more suited for *in vivo* gene therapy applications than 62L-LV (13, 14, 16, 29, 43, 44). Even if CD62L-positive non-T cells will be protected from transduction, they could function as a sink for 62L-LV particles, thereby limiting their availability for on-target transduction. Yet, the tropism of 62L-LV is much more restricted than that of VSV-LV, thus not excluding potential applications upon direct *in vivo* administration.

Given the considerations above, applications of 62L-LV for *ex-vivo* generated CAR T cells are the most realistic option. Currently approved CAR T cell products available in the US and EU markets are manufactured via transduction with VSV-LV or γ-retroviral vectors. According to information provided on the companies’ homepages, between 2 and 5 weeks are required for CAR T cell production and release. To reduce production times, shorter T cell cultivation and expansion could be beneficial. We show here that CAR T cells generated within 3 days of *ex vivo* handling, using 62L-LV or VSV-LV for gene transfer, control the tumor burden in a mouse model. This result is well in line with the previous observation of Ghassemi and colleagues, who have shown that functional CAR T cells cannot be only generated within 3 days, but can also outperform conventionally generated CAR T cells in xenogeneic mouse tumor models (45). In difference to the published results, we stimulated our cells with IL-7 and IL-15 instead of IL-2, activated the PBMC for only 2 days with αCD3 and αCD28, and administered the cells 24 hours after vector incubation.

While shortening the manufacturing time for CAR T cells appears feasible and desirable, certain safety concerns arise with this procedure. During conventional CAR T cell manufacturing, transduced cells undergo several washing and expansion steps reducing the amounts of residual vector particles to negligible concentrations. In contrast, it can be assumed that particle uptake and gene transfer are not completed for CAR T cell products injected as early as 24 or 48 hours after vector incubation. Vector particles still bound to the T cells may transduce to other cells upon infusion. This risk is expected to be higher for VSV-G pseudotyped vectors with their broad cell tropism than for 62L-LV. Yet, there is a need to ensure that tumor cells are not transduced to avoid CAR epitope masking. The fatality of such a scenario was demonstrated in 2018 in a clinical trial investigating the CAR T cell product Kymriah. In this trial, an accidental transfer of a CD19-CAR into a single leukemic cell during manufacturing led to the relapse and death of a patient (46). The causative for this event was that a CAR construct present in tumor cells can bind in cis to the CAR-specific epitope on the surface of the tumor cell. In this case, CD19 masked the epitope from recognition by CAR T cells, conferring resistance to the CAR T cell product and enabling its proliferation. In order to reduce this potential safety concern, the exact time-point of completed transduction after short-term incubation should be investigated and additional washing steps could be implemented to remove residual particles from the cells prior to adoptive transfer. Beyond that, selecting a CD62L-negative tumor entity, possibly solid cancer, solves this issue for 62L-LV but not for VSV-LV.

Recently, rigorous characterization of enriched CAR T_scm_ cells revealed a unique ability to counteract leukemia re-challenge and lower risks for CAR T cell-induced cytokine release syndrome but also a slightly reduced cytotoxic potential compared to conventional CAR-T cells (47). The latter finding is well conceivable given that CAR T_eff_ cells are most active in tumor cell killing but depleted after enrichment for more naïve T cells. It is, moreover, well in agreement with our observation that the cytolytic activity of CAR T cells generated with 62L-LV was less pronounced than that of CAR T cells generated with VSV-LV. Yet, *in vivo*, these less differentiated CAR T cells controlled tumor growth similarly well as CAR T cells generated by VSV-LV, while exhibiting a tendency to be less differentiated and exhausted.

While so far no immediate therapeutic advantage of 62L-LV over VSV-LV has become apparent, it is likely that this will become evident in future studies. For example, re-challenge experiments in a similar mouse setting as the one described here will reveal if CAR T cells generated with 62L-LV are less exhausted and accordingly more potent upon repeated antigen exposure. In addition to the prophylactic setting we used here, CAR T cells generated with 62L-LV will have to be investigated in a therapeutic setting with established tumor cells before infusion of the vector-cell mix. Along this line, CD34^+^ stem cell humanized mouse models offer the potential to investigate short-term generated CAR T cells within a xenoreaction-free and quiescent immunological surrounding which better mimics the human situation.

Taken together, the newly established 62L-LV offers great potential for the *ex vivo* generation of less differentiated CAR T cells without the need for prior or later T cell subtype selection, while exhibiting increased safety with respect to the transduction of cancer cells. It is thus a suitable alternative to VSV-G pseudotyped LV vectors. One immediate application is its use for short-term generated CAR T cells, which may substantially simplify CAR T cell production. Although promising, this approach will need further investigation with regard to safety concerns and scalability of vector production before being implemented into clinical studies.

## Materials and methods

### Ethics statement

Work performed with primary cells isolated from blood donations was invariably obtained from anonymous donors that had provided written informed consent in full compliance with the requirements of the Ethics Committee of the University Hospital Frankfurt, Germany.

### Cell lines and primary cells

HEK293T (ATCC CRL-11268), HT1080 (ATCC CCL-121), and HT1080_αHis_ (25) cells were cultivated in DMEM (Sigma-Aldrich, Munich, Germany) and supplemented with 10% fetal calf serum (FCS; Biochrom, Berlin, Germany) and 2 mM L-glutamine (Sigma-Aldrich, Munich, Germany). The culture medium of HT1080_αHis_ cells was furthermore supplemented with 1.2 mg/mL G418 (Thermo Fisher Scientific, Darmstadt, Germany). The cell line HT1080_CD62L_ was generated by transduction of the parental HT1080 cell line with LV particles encoding the CD62L receptor (UniProt: P14151), an internal ribosome entry site (IRES) element and a puromycin resistance gene under control of the spleen focus-forming virus (SFFV) promoter followed by a woodchuck posttranscriptional regulatory element (WPRE) (transfer plasmid: pS-CD62L-IPW). Transduced cells were selected using puromycin for 2 weeks. Nalm-6-eBFP-Luc (kindly provided by Prof. Helen Fielding, University College of London), further on called Nalm6, were grown in complete Roswell Park Memorial Institute (RPMI) medium (RPMI 1640, Biowest) and supplemented with 10% FCS and 2 mM L-glutamine.

Human PBMC were isolated from fresh blood of healthy donors or buffy coats purchased from the German blood donation center (DRK-Blutspendedienst Hessen, Frankfurt) and cultured in T cell medium (TCM), consisting of RPMI 1640 supplemented with 10% FCS, 2 mM L-glutamine, 0.5% streptomycin/penicillin, and 25 mM HEPES (Sigma-Aldrich, Germany) or 4Cell^®^ Nutri-T medium (Sartorius, Germany) supplemented with 0.5% streptomycin/penicillin and in the presence of 25 U/mL IL-7 and 50 U/mL IL-15 (all cytokines from Miltenyi Biotec, Germany). For activation, 1x10^7^ PBMC per 6-well were cultured in TCM supplemented with 3 μg/mL anti-CD28 antibody (clone 15E8, Miltenyi Biotec, Germany) for 48 hours or 72 hours for the CD62L cell separation experiment and *in vitro* cytotoxicity assay. Well plates for activation were pre-coated with 1 µg/mL anti-CD3 antibody (clone OKT3, Miltenyi Biotec, Germany).

### Generation of CD62L-targeted envelope constructs

To generate the CD62L-targeting constructs, the coding sequences of the variable light chain (VL) and heavy chain (VH) of the parental CD62L-specific monoclonal antibody 145/15 were synthesized *de novo* (GeneArt, Thermo Fisher Scientific) and cloned into the backbone encoding the modified Nipah virus (NiV) glycoprotein G with and without glycine-serine linker(18) (pCG-G_NiV_Δ34mut-His and pCG-G_NiV_Δ34mut-L3-His) or the modified measles virus (MV) hemagglutinin protein (pCG-H_MVnse_Δ18mut-L3-His) (24) via digestion with *Sfi*I and *Not*I. DNA sequences were verified by standard sequencing technologies prior to use in LV production.

### LV production and characterization

Here, we used a second-generation vector platform to show proof of principle for CD62L-targeted LVs. However, transferring our paramyxovirus-based vector targeting system to a third-generation vector platform has already been shown to be feasible (48, 49). Vector particles were generated by transient transfection of adherent HEK-293T cells using polyethylenimine (PEI) and second-generation packaging plasmids as described in detail by Weidner and colleagues (49). In brief, 1 day before transfection, 1.5-2x10^7^ cells were seeded into a T175 flask. In total, 35 μg DNA was added to 2.3 mL of DMEM without additives and combined with 2.2 mL DMEM containing 140 μL of 18 mM PEI solution. The transfection solution was mixed and incubated for 20 min at room temperature. The cell medium was replaced by 10 mL DMEM supplemented with 15% FCS and 3 mM L-glutamine before the transfection solution was added to HEK-293T cells. The medium was replaced by DMEM with 10% FCS and 2 mM L-glutamine 4-6 hours later. Two days after transfection, the cell culture supernatant was collected and filtrated. Alternatively, vector particles were generated by transient plasmid transfection of 5x10^7^suspension HEK-293 cells using the LV-MAX™ lentiviral production system (Thermo Fisher Scientific). In brief, on the day of transfection, suspension cells were seeded at 4.7x10^6^ cells/mL in LV-MAX production medium and 59 µL LV-MAX supplement was added per mL cell suspension. For transfection, 2.5 µg DNA was used per mL cell suspension diluted in Opti-MEM and incubated with diluted transfection reagent (6 µL/mL cell suspension) for 10 minutes at room temperature. Subsequently, the DNA-lipid complex was added to the cells. 40 µL LV-MAX enhancer per mL cell suspension was added 5 – 14 hours later. Two days post-transfection, vectors were harvested by pelleting cells (3 minutes, 300 g) and the supernatant was collected which was filtered through a 0.45 µm filter. Released vector particles were concentrated over a 20% sucrose cushion at 4500xg for 24 hours before the supernatant was discarded and pellets were resuspended in 60 µL PBS. The used transfer plasmid encoded a second-generation CD19-CAR in conjunction with ΔLNGFR (21). Notably, based on the co-expression of ΔLNGFR and the CAR construct, the detection of ΔLNGFR can be used as a surrogate marker for the expression of CAR molecules on the cell surface. Plasmid ratios for the generation of NiV-based and MV-based RT-LV particles as well as particles pseudotyped with VSV-G were described previously (18, 44) and can be found in **Supplementary Tables 1, 2**. If not otherwise specified, all concentrated vector stocks were titrated on HT1080_αHis_ cells as described previously using an LNGFR-specific antibody for detection (21). LV particle yields were determined by nanoparticle tracking analysis or p24-specific enzyme-linked immunosorbent assay (HIV type 1 p24 Antigen ELISA; ZeptoMetrix Corporation) according to the manufacturer’s instructions and calculated as described (18, 21).

### Transduction of cell lines and primary cells

Parental HT1080, HT1080_αHis_, and HT1080_CD62L_ cells were seeded at 8x10^3^ cells per 96-well and incubated with serial dilutions of vector stocks. Transgene expression was analyzed 72 to 96 hours later by flow cytometry. Activated PBMC were seeded at 4x10^4^ or 8x10^4^ cells per 96-well, respectively, in TCM medium before CD62L-LV (5 µL or 10 µL) or VSV-LV (0.05 µL or 0.5 µL) were added. Where indicated, CD62L-LV transduction of PBMC was carried out in the presence of Vectofusin-1 (Miltenyi Biotec, Germany) as described previously (21). Cells were centrifuged at 850g and 32°C for 90 minutes, followed by the addition of TCM supplemented with cytokines. The medium was replenished every 2 to 3 days. Optionally, cells were passaged. Transgene expression was assessed by flow cytometry.

### Quantification of shed CD62L by ELISA

Activated PBMC of three donors were cultured without medium change or cell passaging for up to 10 days. At the indicated time points, the cell suspension was collected and centrifuged for 5 minutes at 5,000 rpm and either stored at -80°C or 4°C. The concentration of sCD62L in the supernatant was determined by ELISA (Human L-Selectin/CD62L DuoSet ELISA, R&D Systems) following the manufacturer’s protocol with the exception that heat-inactivated FBS was used instead of inactivated goat serum. Quantification of the fluorescent signals was performed with a microplate reader (EmaxPlus, Molecular Devices).

### Blocking assay with shed CD62L or antibodies

For the blocking assay with antibodies, 4x10^4^ activated PBMC were preincubated with the indicated concentrations of a CD62L-specific antibody (clone 145/15, Miltenyi Biotec) or a CD45-specific antibody (clone 5B1, Miltenyi Biotec) either conjugated to the fluorophore phycoerythrin (PE)-Vio770 or to biotin for 1 h at 4°C. Before and after antibody incubation, cells were washed twice with wash buffer (phosphate-buffered saline (PBS) supplemented with 2% FBS and 0.1% NaN_3_). Afterward, either 10 µL of 62L-LV or PBS was added to cells pre-incubated with biotin-conjugated antibodies, while PBS was added to cells pre-incubated with fluorophore-conjugated antibodies. All samples were incubated at 4°C for 30 min. Cells pre-incubated with biotin-conjugated antibodies were further stained with a PE-labeled anti-LNGFR antibody (clone ME20.4-1.H4, Miltenyi Biotec). After two additional washing steps, antibody and vector-bound cells were determined by flow cytometry analysis.

For the sCD62L blocking assays, 10 µL of 62L-LV or VSV-LV vector particles was pre-incubated with 90 µL fresh or frozen supernatant containing sCD62L derived from 6 days of PBMC culture or TCM only for 1 h at 4°C. Vector/sCD62L-containing supernatant was then added to 4x10^4^ activated PBMC of various donors in 96 wells. Staining for vector-bound cells was performed after incubation for 30 minutes at 4°C by flow cytometry detecting ΔLNGFR.

### CD62L cell separation

Activated T cells were labeled with cleavable CD62L-APC (clone REAL163), bound to paramagnetic anti-APC microbeads, and separated via magnetic-activated cell separation into CD62L- and CD62L+ cells according to the manufacturer’s instructions of the Anti-APC Microbeads Kit (Miltenyi Biotec, Germany). To release the CD62L-APC antibody for transduction experiments, it was enzymatically cleaved using the REAlease^®^ Support Kit (Miltenyi Biotec, Germany) according to the manufacturer’s instructions. CD62L expression on separated cells was determined by flow cytometry detecting CD62L-APC labeling or additional staining with CD62L-PE-Vio770 (clone 145/15). All antibodies were from Miltenyi Biotec (Bergisch Gladbach, Germany).

### Animal experiment

All animal experiments were conducted in accordance with the German Animal Protection Law and the respective European Union guidelines.

For the short-time generation of CAR T cells, 1.8x10^6^ activated PBMCs were seeded in 600 µl TCM per 24-wells, mixed with 30.6 µl 62L-LV (equals MOI of 1.3 or ~4x10^10^ vector particles) or VSV-LV (equals an MOI of 8.8 or ~3x10^10^ vector particles) or equal volume of PBS and centrifuged for 90 min at 850g and at 32°C before the addition of TCM to a total volume of 1.2 mL per well. Detailed vector parameters of the used 62L-LV and VSV-LV stock can be found in **Supplementary Table 3**. Prior to *in vivo* application, cells were harvested and washed 2x with PBS 24h after vector incubation. NSG mice (NOD.Cg.Prkdc^scid^IL2rg^tmWjl^/SzJ, Jackson Laboratory) were intravenously (i.v.) injected with 2x10^6^ vector-bound cells or 1.4x10^6^ PBS-treated cells. Three days later, 5x10^5^ Nalm-6 was injected i.v. and tumor growth was monitored by bioluminescence imaging (BLI). This was performed by injecting D-luciferin (Perkin Elmer) intraperitoneally at 150 µg/kg body weight and imaging luciferase signals 10 minutes after injection using the IVIS Imaging System (Perkin Elmer). CAR T cell engraftment was monitored through regular blood drawings. Mice were checked regularly for health status and tumor load by IVIS. All mice were sacrificed on day 17 for the final analysis of blood and organs (spleen, bone marrow, and liver).

Collected blood and organs were prepared to a single cell suspension and analyzed by flow cytometry analysis. Blood was washed with PBS prior to and after erythrocyte lysis using BD Pharm Lyse buffer (BD Bioscience). Spleens were minced through a 70 µm cell strainer to obtain a single-cell solution and then subjected to erythrocyte lysis. Bone marrow was harvested through centrifugation of long bones cut open with a scalpel in pierced 0.5 mL tubes at 8000 rpm for 5 min. Bone marrow cells were then washed with PBS and singularized through a 70 µm cell strainer and erythrocyte lysis was performed. Liver cells were isolated using the mouse liver dissociation kit (Miltenyi Biotec) according to the manufacturer’s instructions, washed with PBS, and erythrocytes were lysed.

### Flow cytometry

Flow cytometry analysis was performed using MACSQuant Analyzer 10 (Miltenyi Biotec, Bergisch Gladbach, Germany) or LSR Fortessa (BD Biosciences) flow cytometers. Data were analyzed by FCS Express 6 (*De Novo* Software, Glendale, CA, USA) or FlowJo 7 (BD Biosciences). Before and after staining with fluorescently labeled antibodies, cells were washed twice with wash buffer. Before measurement, cells were fixed by the addition of PBS supplemented with 1% formaldehyde. To determine the percentage of transduced cells or cell-bound vector particles, staining of up to 1x10^5^ cells was performed. The reporter protein ΔLNGFR, which is co-expressed with the CD19-CAR, was detected using the anti-LNGFR-PE antibody. PBMC were further stained with the fixable viability dye eFluor780 (Life Technologies, Darmstadt, Germany), according to the manufacturer’s instructions, or with 7-AAD to detect viable cells. To further characterize the PBMC, cells were stained in addition with a CD4-specific antibody (clone VIT4) labeled with VioGreen or PE-Vio770 and a CD8-specific antibody (clone BW135/80) labeled with allophycocyanin (APC) or APCVio770 and if indicated with a CD62L-specific antibody (clone 145/15), labeled with PEVio770. All antibodies were from Miltenyi Biotec (Bergisch Gladbach, Germany). The following antibodies were used for flow cytometry analysis of the *in vivo* experiment: CD45-BV510 (clone 2D1, BioLegend), CD3-BV605 (clone HIT3a, BD Bioscience), CD8-BV786 (clone RPA-T8, BD Bioscience), LNGFR-PE (clone ME20.4-1.H4, Miltenyi Biotec), CD4-PE-CF594 (clone RPA-T4, BD Bioscience), CD19-Alexa Fluor 700 (clone HIB19, Thermo Fisher), TIM-3-FITC (clone F38-2E2, Miltenyi Biotec), LAG-3-Alexa Fluor 647 (clone T47-530, BD Bioscience), and eFluor780 (eBioscience). Representative gating strategies can be found in the **Supplementary Material** (**Supplementary Figures 8A, 15**).

### Statistical analysis

Statistical analyses were performed with Prism 7 software (GraphPad). Tests for statistical significance used the unpaired or paired two-tailed Student’s t-test, ordinary one-way ANOVA (Dunnett multiple comparisons test), ordinary two-way ANOVA (Dunnett or Turkey multiple comparisons test), RM two-way ANOVA (Šídák’s comparison test), or Fisher’s least significant difference (LSD) test as indicated. Statistical differences in experiments were considered significant at *p* < 0.05.

## Data availability statement

The original contributions presented in the study are included in the article/**Supplementary Material**. Further inquiries can be directed to the corresponding author.

## Ethics statement

The studies involving human participants were reviewed and approved by Ethikkommission des Fachbereichs Medizin der Goethe-Universität. The patients/participants provided their written informed consent to participate in this study. The animal study was reviewed and approved by Regierungspräsidium Darmstadt, Dezernat V54 - Veterinärwesen und Verbraucherschutz.

## Author contributions

LK, NH, TK, and AJ designed and performed the experiments. LK, TK, NH, AF, FT, JH, and CB evaluated the data. TS contributed protocols and reagents and to the writing of the manuscript. CB and JH conceived and designed the study, acquired grants, supervised the work, and wrote the manuscript. All authors contributed to the article and approved the submitted version.
