# Supplementary material for: CD62L as target receptor for specific gene delivery into less differentiated human T lymphocytes
Source: Front Immunol. 2023 Aug 14;14:1183698. doi: 10.3389/fimmu.2023.1183698 (PMC10461316; doi:10.3389/fimmu.2023.1183698)
Supplement: Supplementary file 1 [file DataSheet_1.pdf]

CD62L as target receptor for specific gene delivery into less differentiated human T lymphocytes

Laura Kapitza, Naphang Ho, Thomas Kerzel, Annika M Frank, Frederic B Thalheimer, Arezoo Jamali, Thomas Schaser, Christian J Buchholz, Jessica Hartmann*

*** Correspondence:** Jessica Hartmann, jessica.hartmann@pei.de

Supplemental Data

In vitro Cytotoxicity assay

CAR T cells generated by 62L-LV or VSV-LV or untransduced T cells were co-cultivated at an effector:target ratio of 0.2:1 with $1x10^4$ cell-trace-violet labeled Nalm6 cells in 4Cell® Nutri-T medium supplemented with 0.5% streptomycin/penicillin without cytokine supplementation for 24 hours in 96-well u-bottom plates. CAR specific tumor cell lysis was determined by flow cytometry analysis of dead labeled tumor cells by staining with eFluor780 (eBioscience) and normalization to unspecific tumor cell killing by untransduced T cells. CCR7 (APC, clone REA546, Miltenyi Biotec) and CD45RA (VioBlue, clone T6D11, Miltenyi Biotec) expression were determined for phenotype assessment of CAR T cells (CCR7+/CD45RA+ naïve, stem-cell memory; CRR7+/CD45RA- central memory; CCR7-/CD45RA- effector memory; CCR7-/CD45RA+ effector).

Supplementary Tables

Suppl. Table 1: Plasmid compositions for lentiviral vector productions.

	VSV-LV	62L-LV ($^{MV-L3}$62L-LV)	^{NiV}62L-LV	$^{NiV-L3}$62L-LV
**Packaging plasmid:** pCMVΔ8.91 (Zufferey et al., 1997)	65[a]	51.8	82.5	82.5
**Transfer plasmid:** pSEW(Demaison et al., 2002) or pMB-CD19.CAR(4z) (Jamali et al., 2019)	100	86.7	86.7	86.7
**Envelope plasmid:** pMD2.G (Salmon and Trono, 2007)	35			
pCG-G_{NiV}Δ34mut-scFv.CD62L-His			5.1	
pCG-G_{NiV}Δ34mut-L3-scFv.CD62L-His				5.1
pCG-H_{MVnse}Δ18mut-L3-scFv.CD62L-His		7.7		
pCAGGS-F_{NiV}Δ22 (Bender et al., 2016)			25.6	25.6
pCG-F_{MVnse}Δ30 (Funke et al., 2008)		53.9		

[a]Plasmid amounts for transfection per cm^2 culture area are provided in [ng].

Suppl. Table 2: Plasmid compositions for lentiviral vector production in suspension cells.

	VSV-LV	62L-LV
**Packaging plasmid:** pCMVΔ8.91 (Zufferey et al., 1997)	813[a]	1030
**Transfer plasmid:** pMB-CD19.CAR(4z) (Jamali et al., 2019)	1250	1080
**Envelope plasmid:** pMD2.G (Salmon and Trono, 2007)	438	
pCG-H_{MVnse}Δ18mut-L3-scFv.CD62L-His		96
pCG-F_{MVnse}Δ30 (Funke et al., 2008)		289

[a]Plasmid amounts for transfection per ml cell suspension are provided in [ng].

Suppl. Table 3: Vector stocks applied for CAR T cell generation for the animal experiment

	Titer (t.u./ml)[(o)]	p24 (µg/ml)	particle concentration (number/ml)	particles/cell[(i)]	MOI[(x, i)]
**62L-LV**	7.51×10^7	107.87	1.27×10^{12}	2.16×10^4	1.28
**VSV-LV**	5.20×10^8	86.67	1.02×10^{12}	1.73×10^4	8.84

[(o)] determined on PBMC; [(x)] 30.6 µl vector stock per 1.8×10^6 cells; [(i)] used for transduction of primary T cells in the animal experiment

Supplementary Figures

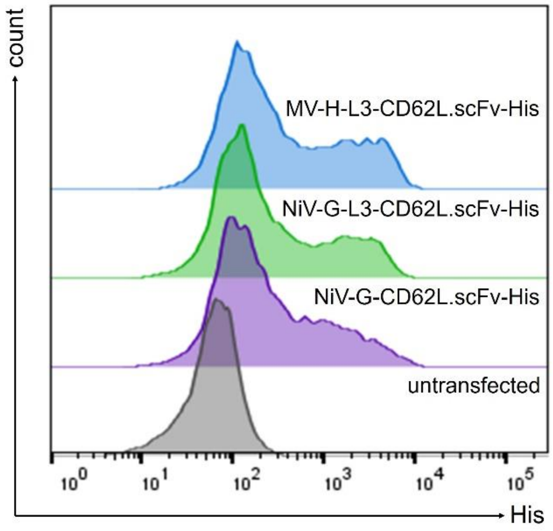

Suppl. Figure 1: Surface expression of glycoproteins fused to the CD62L-scFv.

Producer HEK-293T cells were transiently transfected with plasmids coding for the indicated glycoproteins fused to CD62L-scFv and the His-tag. Surface expression was analyzed three days later. Untransfected HEK-293T cells served as control (grey). All cells were stained with a phycoerythrin labeled αHis antibody. A representative data set is shown.

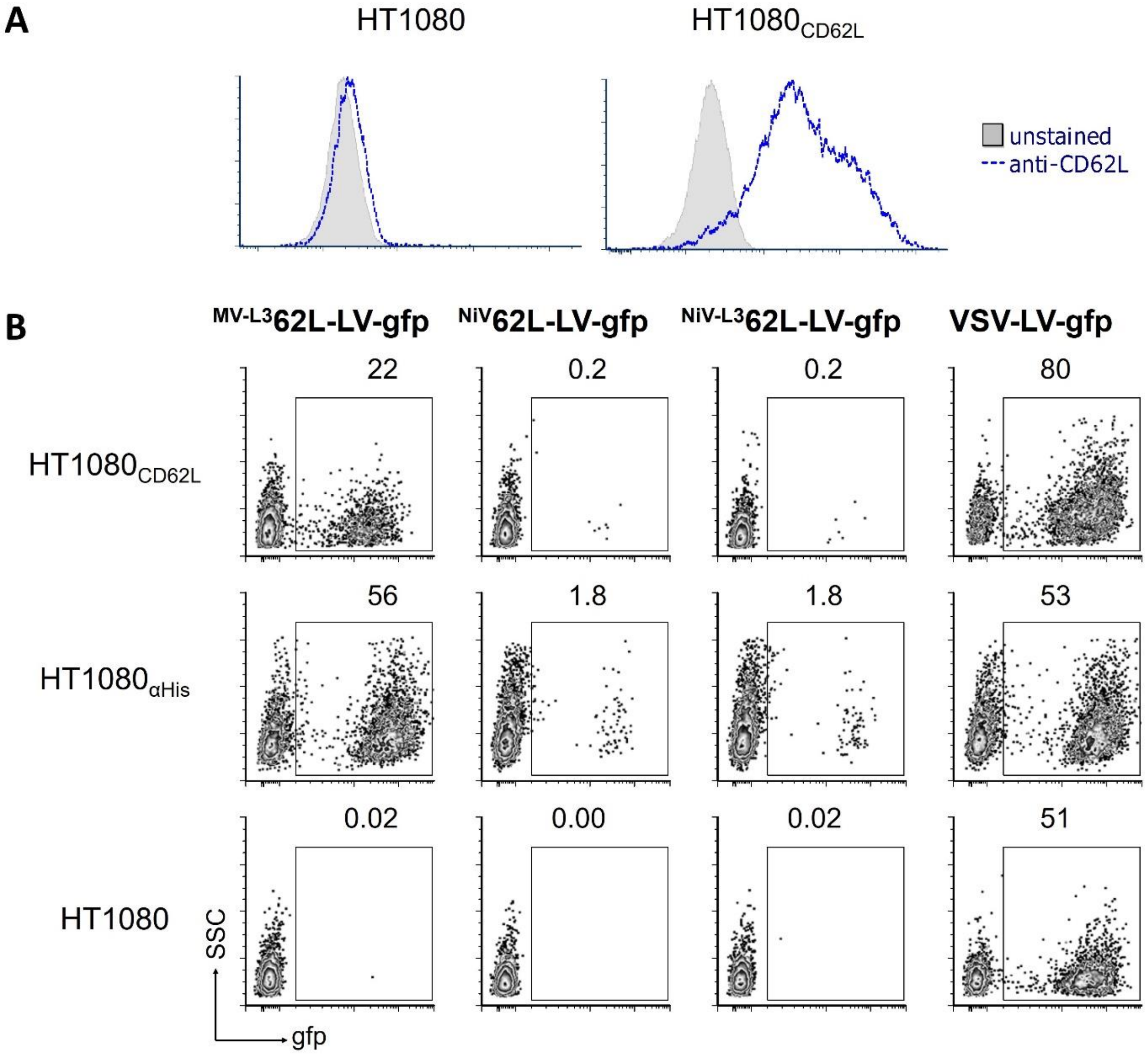

Suppl. Figure 2: Screening CD62L-targeted LVs for transduction efficiency.

A) Parental HT1080 cells and HT1080 cells genetically modified to express human CD62L ($HT1080_{CD62L}$) were stained with a VioBlue labeled αCD62L antibody (blue dotted line) and analyzed by flow cytometry. Unstained cells served as control (grey). **B)** The indicated GFP-encoding vector stocks were produced in HEK293T producer cells in 12-well format as described before by (Hartmann et al., 2018). 100 µL supernatant containing vector particles was used for transduction of the indicated target ($HT1080_{CD62L}$ or $HT1080_{\alpha His}$) and non-target cells (HT1080), respectively. $4x10^5$ cells for transduction had been seeded one day before. Four days post transduction cells were analyzed by flow cytometry for GFP expression (x-axis). One representative data set is shown.

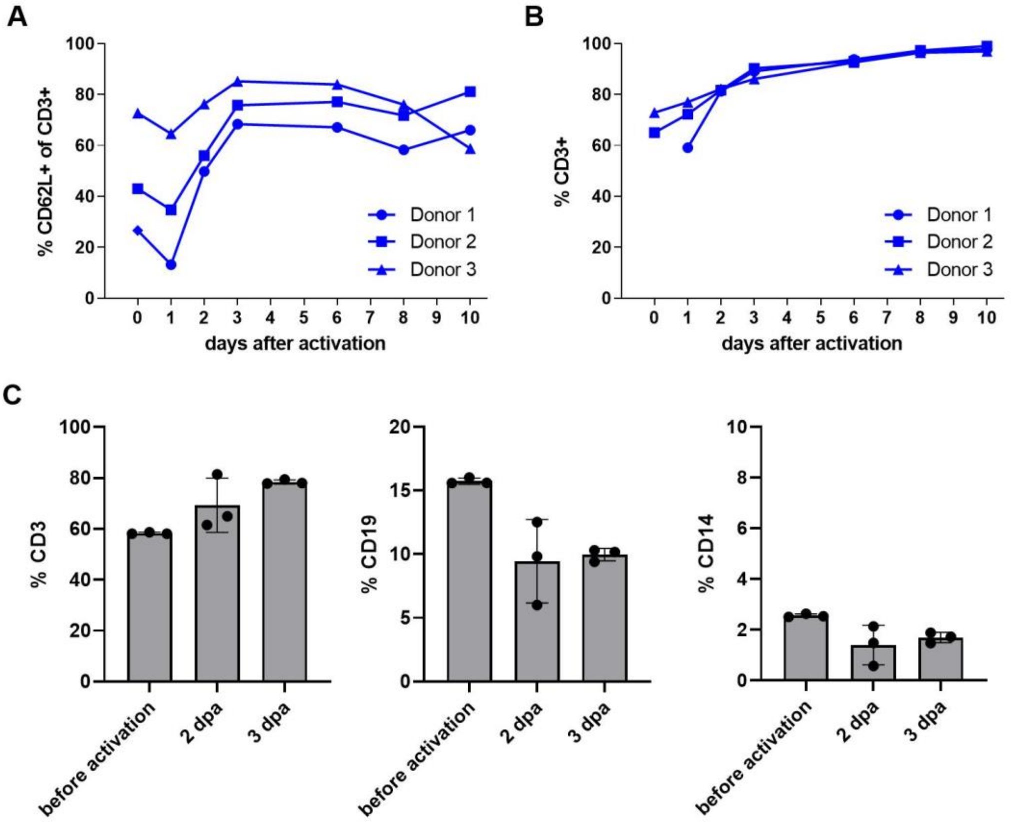

Suppl. Figure 3: Monitoring CD62L expression in cultivated PBMC.

A/B) PBMC from three different donors were isolated and activated before they were stained with a CD3-specific antibody coupled to FITC (clone BW264/56, Miltenyi Biotec) as well as a CD62L-specific antibody (clone 145/15, Miltenyi Biotec) coupled to PE-Vio770 (donor 1) or VioBlue (donor 2 and donor 3). Percentages of CD3-positive cells (B) as well as CD62L-positive cells among CD3-positive cells (A) are reported. Gating was done according to Suppl. Fig. 15. **C)** PBMC from three different donors were isolated and activated. Cells were analyzed for CD3, CD19 and CD14 expression at the day of isolation (before activation) as well as two and three days post activation (dpa) by flow cytometry. Percentages of stained cells are shown. Individual results as well as means with SD are plotted. Gating was done according to Suppl. Fig. 15 with the exception that singlets were not only gated for CD3+ but also for CD19+ and CD14+ cells, respectively.

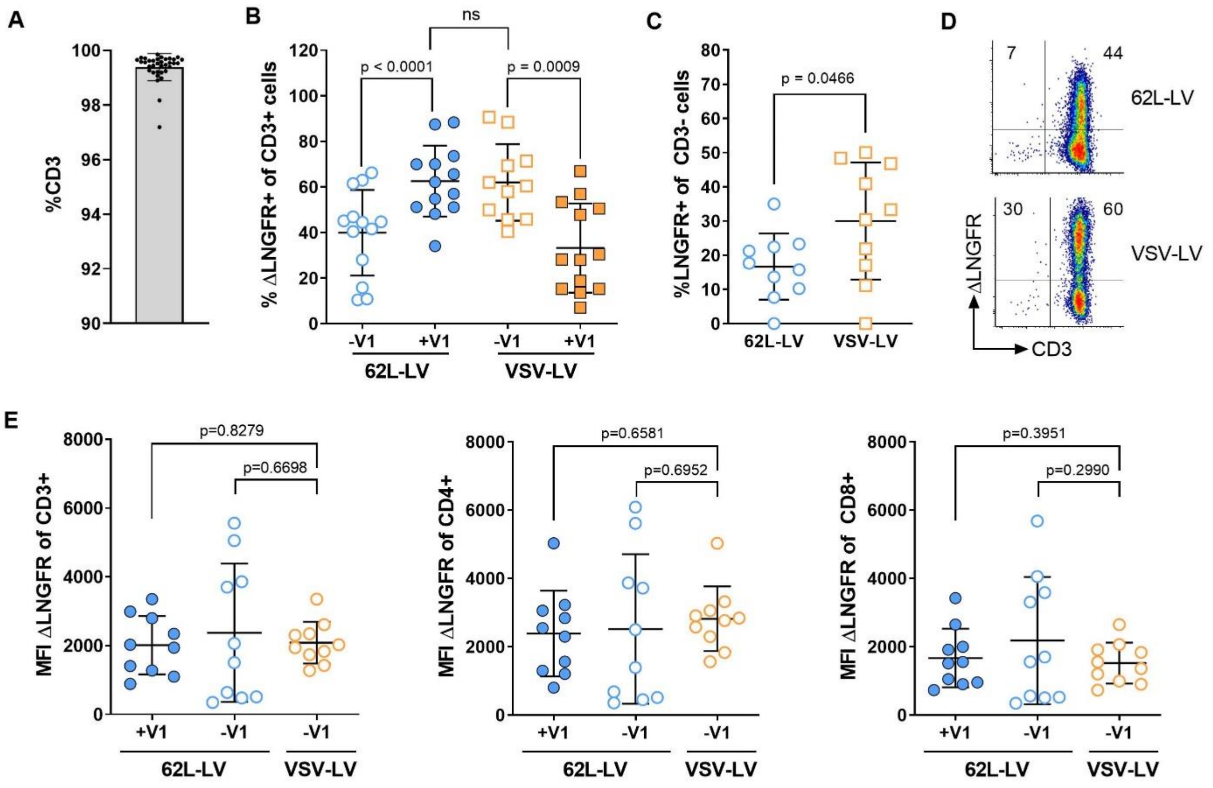

Suppl. Figure 4: Comparison of gene transfer rates into PBMC by 62L-LV and VSV-LV.

4x10^{4} activated human PBMC obtained from seven donors were transduced with 5 µl or 10 µl of 62L-LV or 5 µl VSV-LV and then cultivated in presence of IL-2 or IL-7/IL-15 for 9 to 12 days. Use of the transduction enhancer Vectofusin-1 is indicated by +V1. **A**) Percentages of total CD3-positive cells within the evaluated transduced and untransduced PBMC 9 days post transduction. **B-C**) Flow cytometry was performed to determine the fractions of ΔLNGFR-positive cells on CD3-positive cells (B) and on CD3-negative cells (C). Individual results from four (C) or seven (B) different donors in two (C) or four (B) independent experiments and means with standard deviation (SD) are plotted. Statistical analysis was performed using unpaired t-test. **D**) Representative dot plots of 62L-LV and VSV-LV transduced PBMC. Numbers in the individual gates refer to the percentage of ΔLNGFR-positive cells among CD3-negative cells (upper left gates) and CD3-positive cells (upper right gates), respectively. **E**) Median fluorescent intensity (MFI) of ΔLNGFR-positive cells on CD3-positive (left panel), CD4-positive (middle panel) and CD8-positive (right panel) cells. Individual results from five different donors in two independent experiments and means with standard deviation (SD) are plotted. Statistical analysis was performed using unpaired t-test.

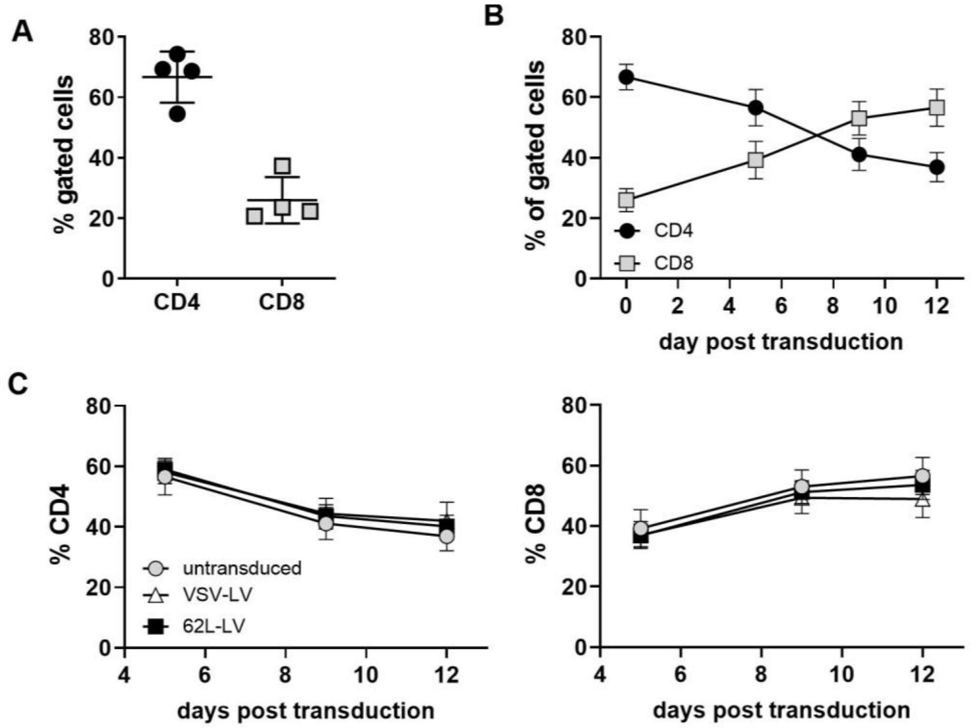

Suppl. Figure 5: Percentages of CD4 and CD8 cells within untransduced and transduced PBMC at various time points

Percentages of CD4+ and CD8+ activated T cells of 4 different healthy donors isolated from buffy coat with or without transduction. Cells were gated according to Suppl. Fig. 15 for the proportion of CD4 and CD8 cells at the indicated time points. **A/B)** CD4 and CD8 percentage of untransduced cells at the day of transduction (day 0) (A) and over a cultivation period of 12 days after transduction (B). **C)** Proportion of CD4 (left) and CD8 (right) cells for untransduced, 62L-LV transduced and VSV-LV transduced PBMC analyzed 5, 9 and 12 days post-transduction.

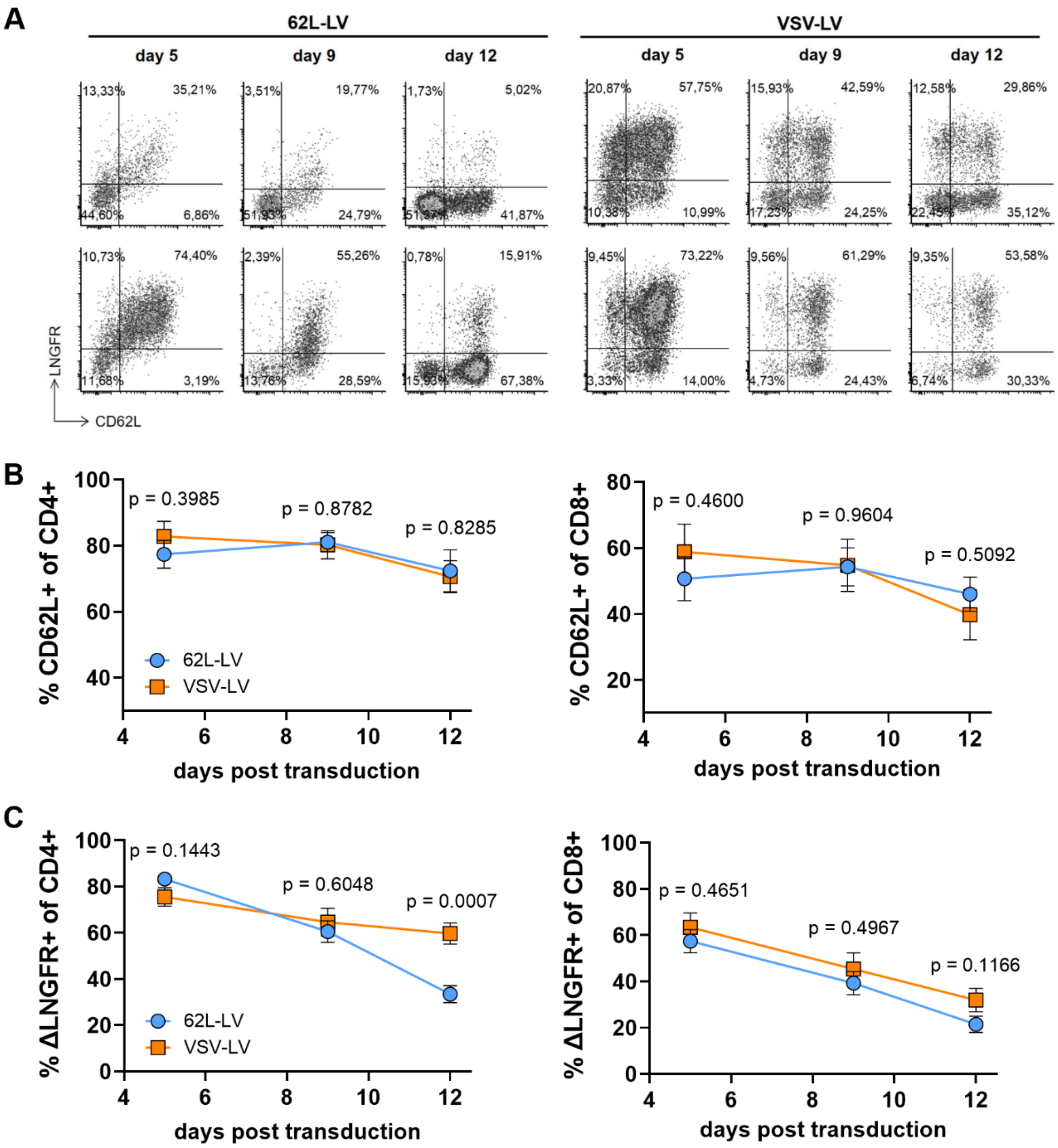

Suppl. Figure 6: Monitoring of cultivated PBMC transduced with 62L- or VSV-LV.

Characterization of activated PBMC incubated with 62L-LV or VSV-LV in the absence of the transduction enhancer Vectofusin-1 (V1) as depicted in Figure 2B. The respective gating strategy can be found in Suppl. Fig. 15. **A)** Representative dot plots of PBMC from one donor 5 days post-transduction with 62L-LV (left) or VSV-LV (right) gated for ΔLNGFR vs. CD62L cells among $CD8^+$ (top) or $CD4^+$ cells (bottom). **B)** The total percentage of CD62L+ cells for the $CD4^+$ (left) and $CD8^+$ (right) fractions over time. **C)** The total percentage of ΔLNGFR+ cells for the $CD4^+$ (left) and $CD8^+$ (right) fractions over time. Statistics and replica are as described for Figure 2B (B-C).

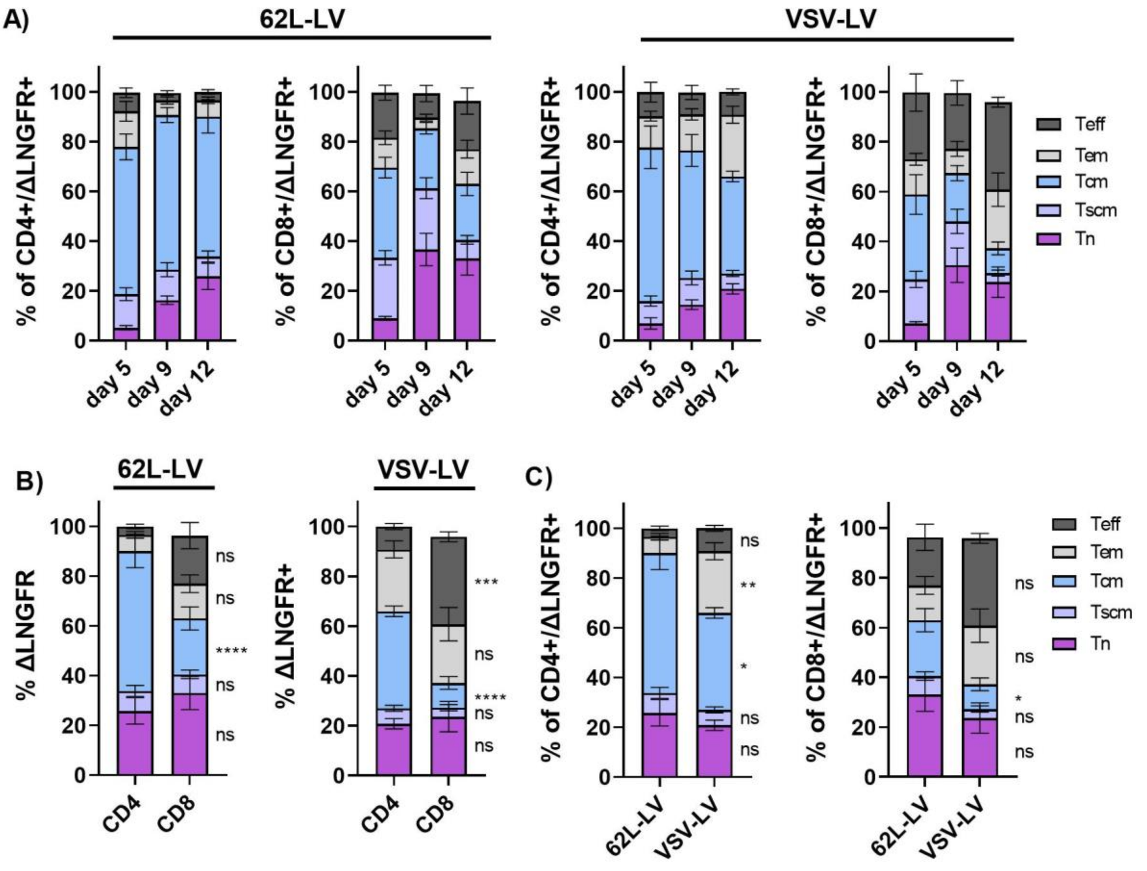

D) **CD4+/ΔLNGFR+ vs. CD8/ΔLNGFR+**

	62L-LV			VSV-LV		
	d5	d9	d12	d5	d9	d12
Teff	0.2626 (ns)	0.5433 (ns)	0.0675 (ns)	0.0182 (*)	0.0566 (ns)	0.0006 (***)
Tem	0.9784 (ns)	0.9891 (ns)	0.5576 (ns)	0.9928 (ns)	0.8146 (ns)	0.9954 (ns)
Tcm	0.0007 (***)	<0.0001 (****)	<0.0001 (****)	<0.0001 (****)	<0.0001 (****)	<0.0001 (****)
Tn	0.9143 (ns)	0.0004 (***)	0.5907 (ns)	>0.9999 (ns)	0.0168 (*)	0.9505 (ns)
Tscm	0.2444 (ns)	0.0623 (ns)	0.9997 (ns)	0.4352 (ns)	0.5752 (ns)	0.9623 (ns)

E) **62L-LV vs. VSV-LV**

	CD4+/ΔLNGFR+			CD8/ΔLNGFR+		
	d5	d9	d12	d5	d9	d12
Teff	0.982 (ns)	0.6527 (ns)	0.7407 (ns)	0.4325 (ns)	0.0835 (ns)	0.1230 (ns)
Tem	0.9895 (ns)	0.3040 (ns)	0.0093 (**)	0.9837 (ns)	0.7336 (ns)	0.2743 (ns)
Tcm	0.9704 (ns)	0.1265 (ns)	0.0157 (*)	0.9805 (ns)	0.8130 (ns)	0.0472 (*)
Tn	0.9909 (ns)	0.9830 (ns)	0.8194 (ns)	0.9883 (ns)	0.6528 (ns)	0.2941 (ns)
Tscm	0.8670 (ns)	0.9905 (ns)	0.9887 (ns)	0.6676 (ns)	0.5500 (ns)	0.8834 (ns)

Suppl. Figure 7: Changes in the differentiation status of CAR+ T cells upon cultivation.

Differentiation status of CD4+ and CD8+ cells expressing ΔLNGFR for the indicated time points. Data belong to the experiment described in Figure 2B. Cells from three different donors transduced with either vector in two individual experiments were analyzed. Mean with standard error (SEM) is plotted. Statistical testing was performed using 2-way ANOVA with Tukey-multiple comparison. **A)** Fractions of Tn, Tscm, Tcm, Tem and Teff among CD4+/ΔLNGFR+ and CD8+/ΔLNGFR+ cells for all analysis time points for 62L-LV (left) and VSV-LV (right). **B/C)** Comparison of CD4+/ΔLNGFR+ and CD8+/ΔLNGFR+ cells for each vector (B) as well as between both vector groups (C) at day 12. **D/C)** Individual p values of statistical testing at the indicated time points. *p < 0.1, **p < 0.01, ***p < 0.001, ****p < 0.0001; ns >0.9999, not significant. The respective gating strategy can be found in Suppl. Fig. 15.

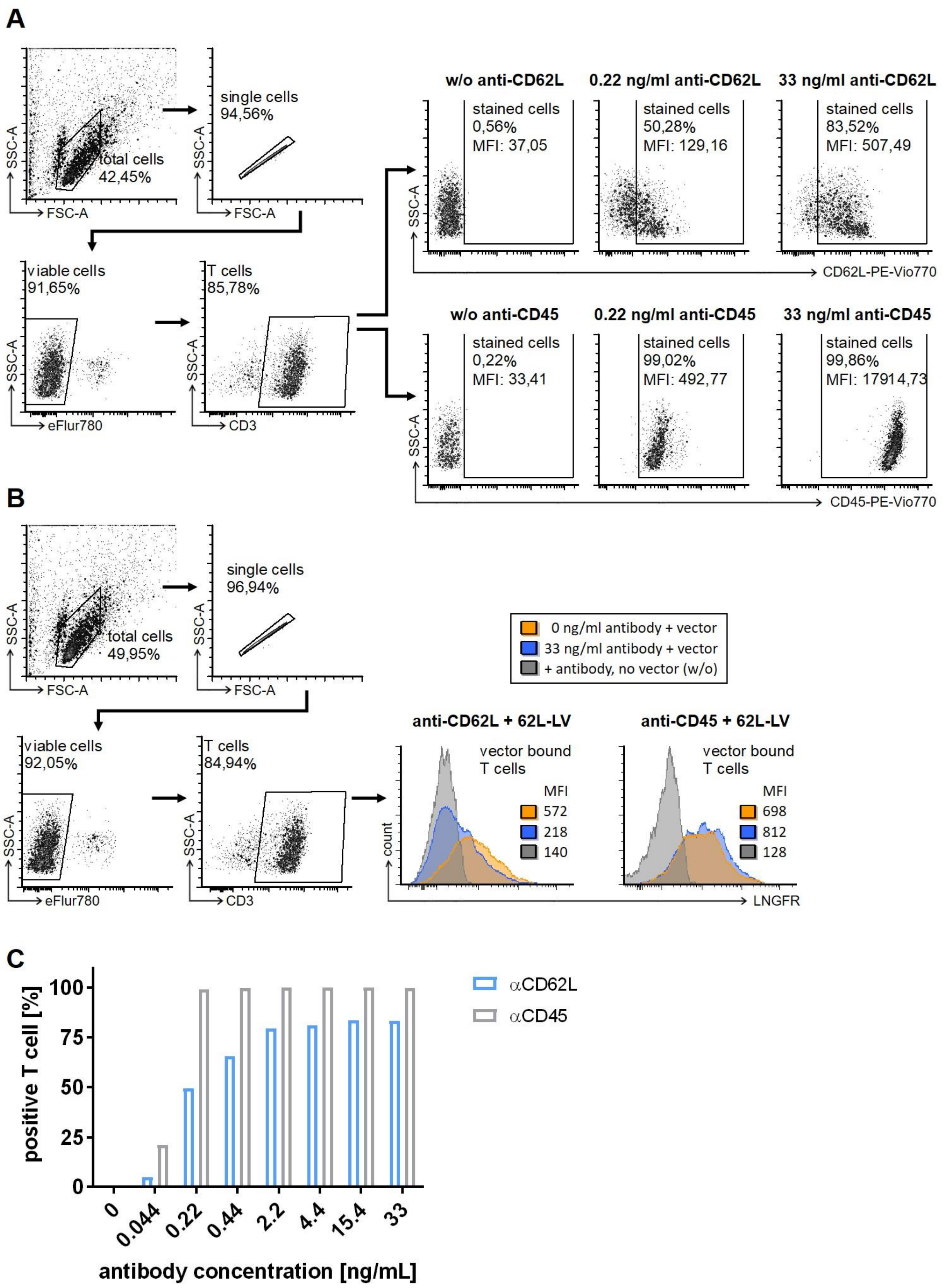

Suppl. Figure 8: Staining capacity of CD62L and CD45 antibodies.

A/B) Gating strategy for antibody binding **(A)** and vector binding **(B)** as depicted in Figure 3 and Suppl. Figure 8C. The cell population is subsequently gated for total cells (PBMC), for singlets, viability, and $CD3^+$ cells. From the $CD3^+$ cell gate cells were gated for CD62L and CD45 antibody binding, respectively **(A)** or analyzed for ΔLNGFR expression **(B)**. **C)** Activated PBMC were incubated either with the parental CD62L-specific antibody clone 145/15 (blue bars) or with a CD45-specific control antibody (clone 5B1, grey bars) at indicated concentrations. Both antibodies were fluorophore-labelled to allow concentration dependent detection of CD62L and CD45. Percentages of stained cells are shown.

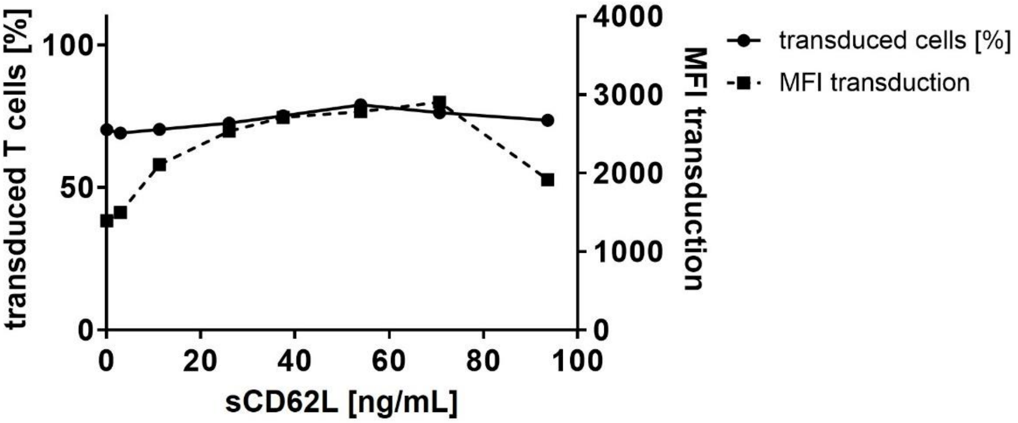

Suppl. Figure 9: Gene transfer activity of 62L-LV in the presence of sCD62L.

PBMC were activated and cultivated in TCM for two days before 62L-LV was added in presence of Vectofusin-1. Directly before transduction $4x10^4$ PBMC were seeded in medium containing the indicated concentrations of sCD62L obtained from a 6-day PBMC culture. Flow cytometry analysis was performed 5 days after transduction. Percentages of transgene positive T cells (solid line) and the MFI for transgene expression (dotted line) are indicated.

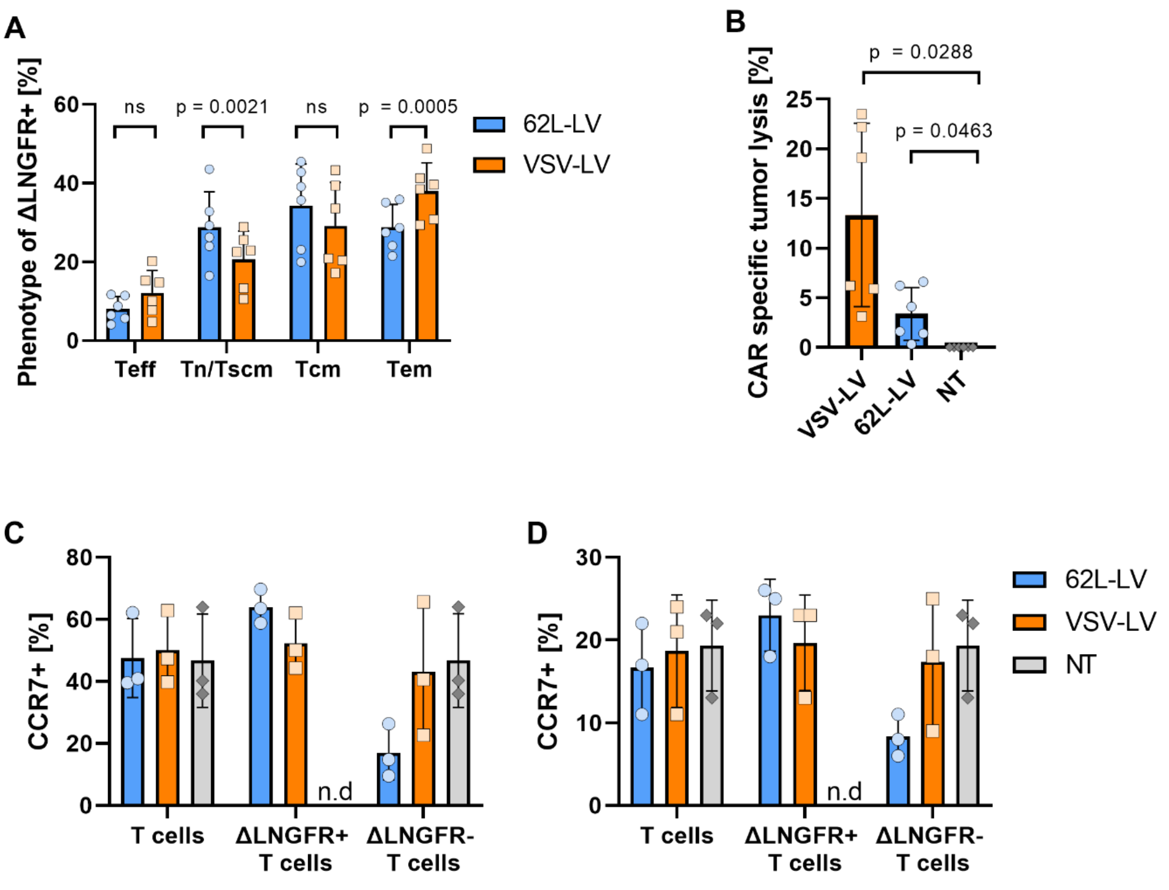

Suppl. Figure 10: *In vitro* cytotoxicity assay.

A) Phenotype of CAR T cells 3 days after transduction with VSV-LV and 62L-LV, determined by CD45RA and CCR7 expression. **B)** CAR-specific tumor lysis of Nalm6 tumor cells. Effector and target cells were co-cultivated for 24 h at a ratio of 0.2:1. Non-transduced cells (NT) were used as control. **C/D)** CCR7 expression on all T cells, ΔLNGFR+ or ΔLNGFR- T cells before (C) and after (D) tumor cell killing. Individual results as well as means with standard deviation with 6 donors (A/B) and with 3 donors (C/D) measured in technical triplicates are shown, respectively. Statistical testing was performed by two-way ANOVA with Šídák’s multiple comparison test (A/C/D) or with RM one-way ANOVA with Dunnett’s multiple comparison (B). No significant differences between 62L-LV and VSV-LV were observed in (C) and (D). n.d (not determined); ns (not significant).

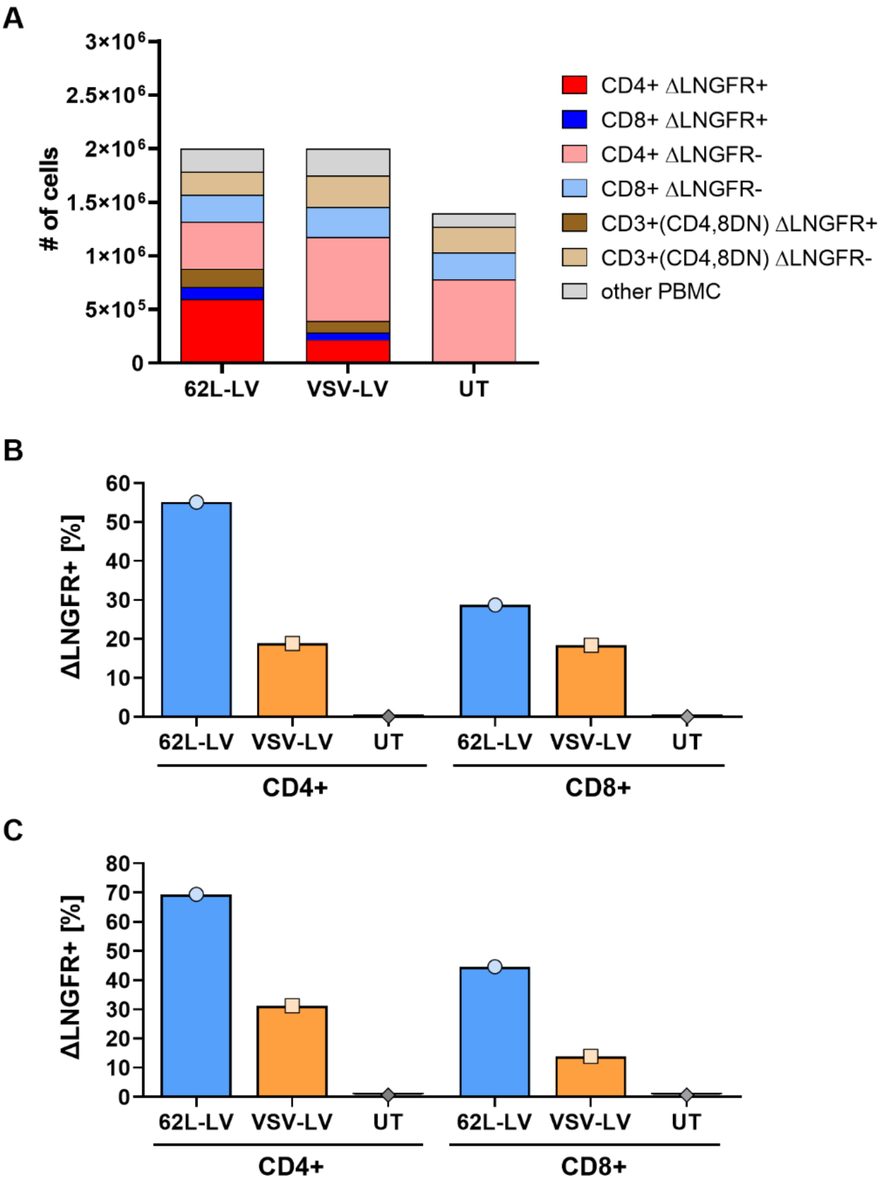

Suppl. Figure 11: Characterization of injected cells.

The composition of the infused cell-vector-mixtures was analyzed by flow cytometry on the day of infusion (day 1; **A/B**) and after 2 days of additional culture (day 3, **C**). Vector bound-cells (**A/B**) and transduced cells **(C)** were identified by staining with an αLNGFR antibody via flow cytometry. **A)** Number of cells injected into individual mice. T cells identified being ΔLNGFR positive or negative or non-T cells being CD3 negative are indicated in different colors. DN = double negative. Percentage of vector bound cells **(B)** or CAR+ T cells **(C)** among $CD4^+$ or $CD8^+$ T cells, which were pre-gated for $CD3^+$ cells.

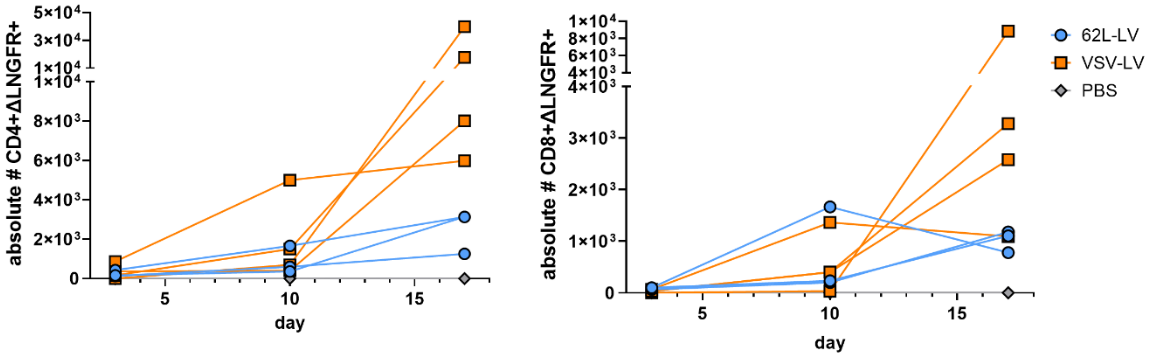

Suppl. Figure 12: CAR T cell numbers in blood.

Absolute numbers of CD4^{+} ΔLNGFR+ (left) or CD8^{+} ΔLNGFR+ (right) cells are shown for each mouse individually at indicated time points. Data belong to the experiment described in Figure 7.

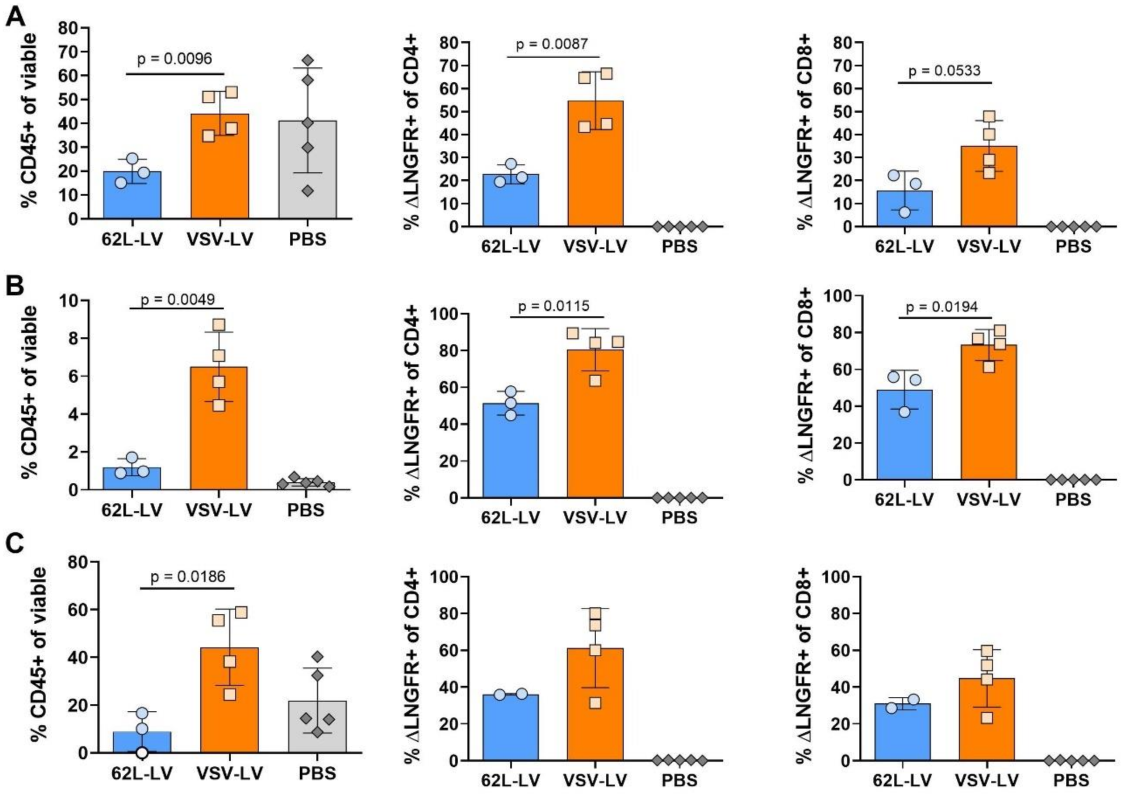

Suppl. Figure 13: Human CD4+ and CD8+ T cells in organs.

Presence of human CD45+ cells (left), ΔLNGFR+ human CD4+ (middle) or ΔLNGFR+ human CD8+ T cells (right) in spleen **(A)**, bone marrow **(B)** and liver **(C)** as determined by flow cytometry at final analysis. Individual results and mean with standard deviation (SD) are depicted. Unpaired t-tests were performed to determine statistics. P values are indicated when below 0.05. Data belong to the experiment described in Figure 7.

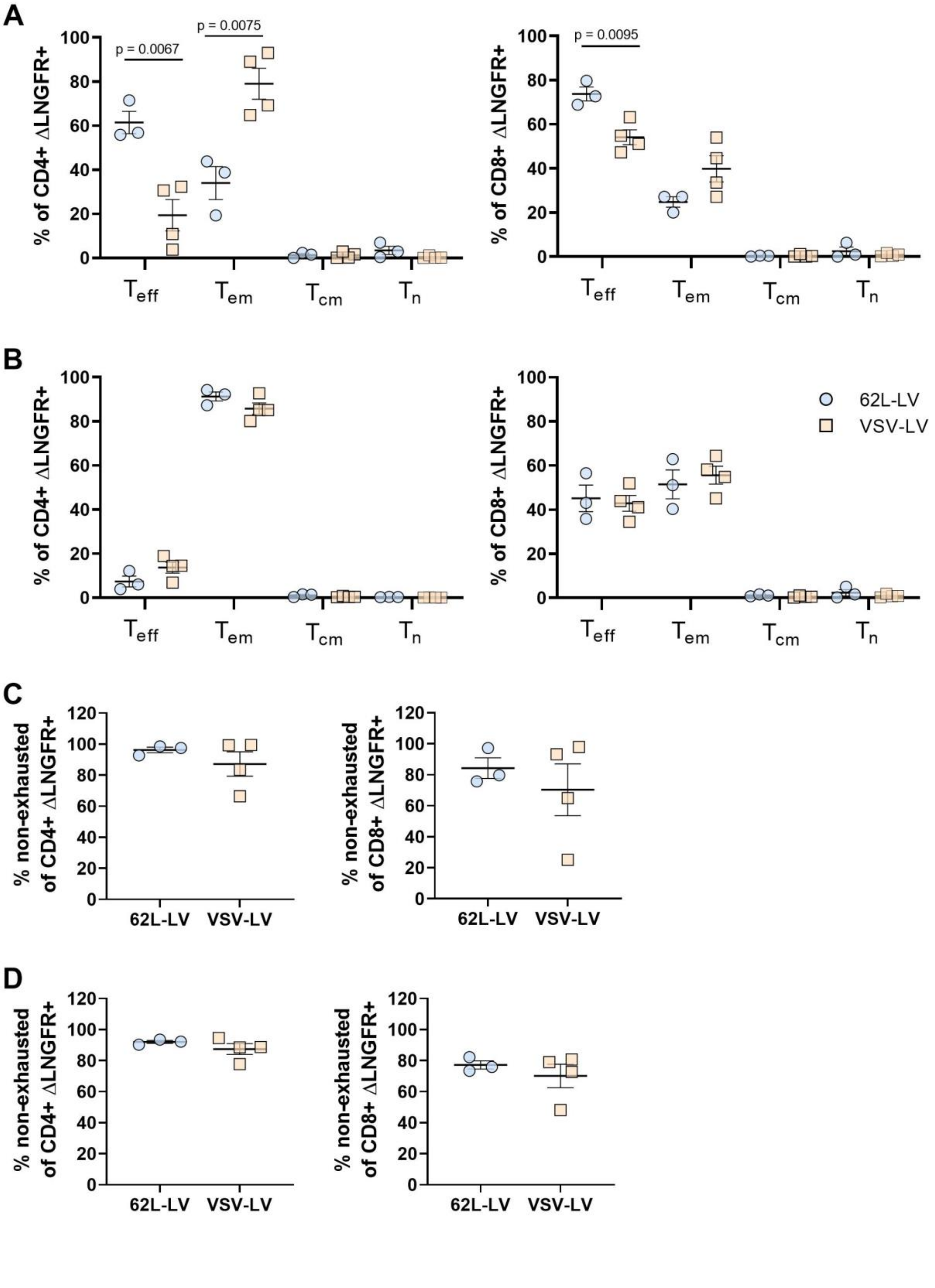

Suppl. Figure 14: Phenotype and exhaustion status of human LNGFR+ T cells in organs.

Frequencies of Teff, Tem, Tcm, Tn of ΔLNGFR+ human CD4+ (left) or ΔLNGFR+ human CD8+ (right) in spleen **(A)** and bone marrow **(B)** as determined by flow cytometry at final analysis. Frequencies of non-exhausted ΔLNGFR+ human CD4+ (left) or ΔLNGFR+ human CD8+ (right) in spleen **(C)** and bone marrow **(D)** as determined by double negative TIM-3 and LAG-3 expression in flow cytometry at final analysis. Individual results and mean with standard deviation (SD) are depicted. Unpaired t-tests were performed to determine statistics. P values are indicated when below 0.05. Data belong to the experiment described in Figure 7.

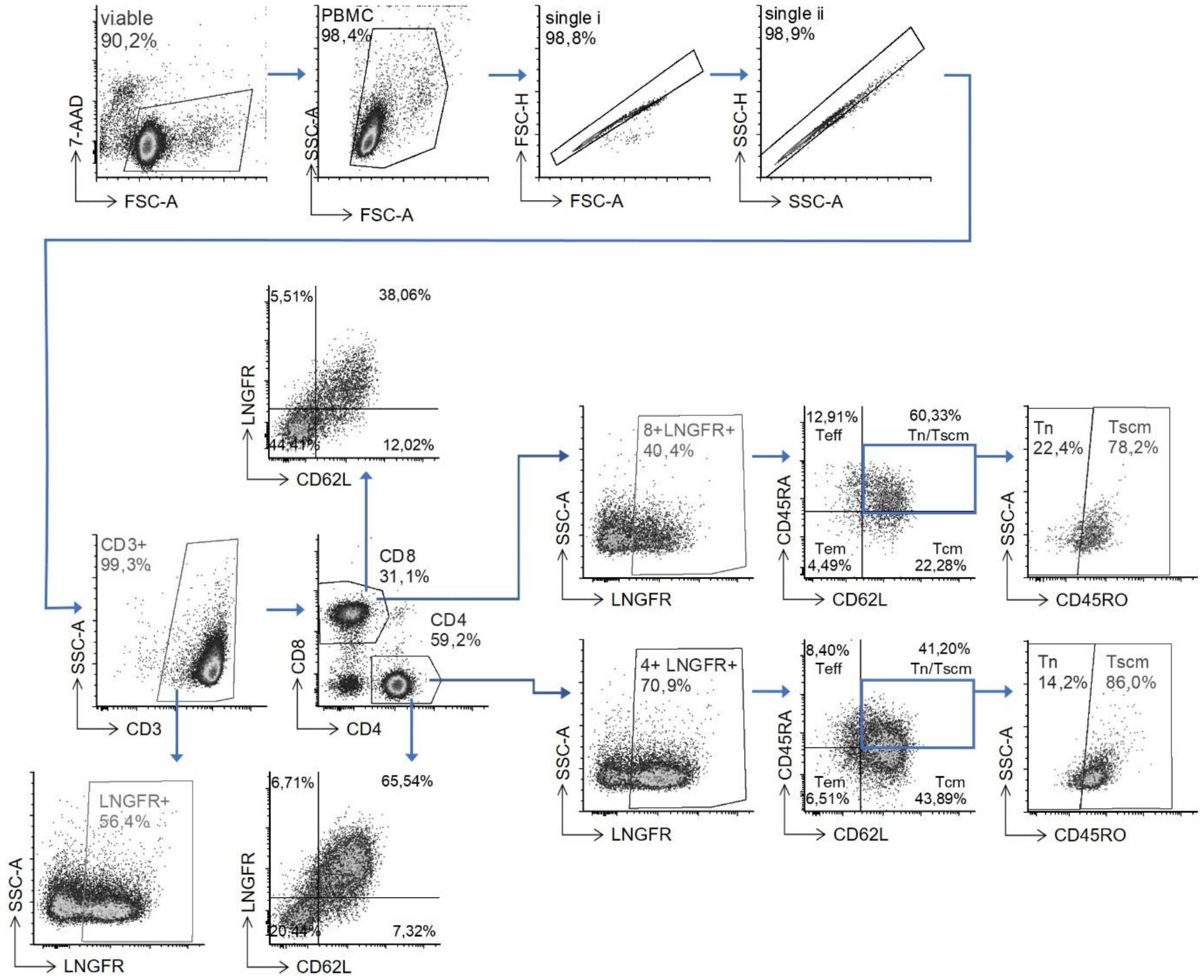

Suppl. Figure 15: Gating strategy for CAR gene delivery and T cell phenotyping.

The consecutive gatings are illustrated by the blue arrows on representative dot plots of PBMC from one donor 5 days post-transduction with 62L-LV without Vectofusin. The cell population was gated for viability, total cells (PBMC), twice for singlets and $CD3^+$ cells. From the $CD3^+$ gate, $CD8^+$ and $CD4^+$ cells were gated. From the $CD3^+$, $CD8^+$ and $CD4^+$ gates, ΔLNGFR-positive cells were further gated, respectively (used for CAR expression in PBMC mediated by 62L-LV or VSV-LV as depicted in Figure 2A and Suppl. Figure 4). For phenotyping as depicted in Figure 2 and Suppl. Figure 7, ΔLNGFR-positive cells were further gated for CD45RA vs. CD62L, respectively. To discriminate between T_n and T_{scm} cells, CD45RO was gated.

REFERENCES

Bender, R. R., Muth, A., Schneider, I. C., Friedel, T., Hartmann, J., Pluckthun, A., et al. (2016). Receptor-Targeted Nipah Virus Glycoproteins Improve Cell-Type Selective Gene Delivery and Reveal a Preference for Membrane-Proximal Cell Attachment. *PLoS Pathog* 12, e1005641. doi: 10.1371/journal.ppat.1005641

Demaison, C., Parsley, K., Brouns, G., Scherr, M., Battmer, K., Kinnon, C., et al. (2002). High-level transduction and gene expression in hematopoietic repopulating cells using a human immunodeficiency virus type 1-based lentiviral vector containing an internal spleen focus forming virus promoter. *Hum Gene Ther* 13, 803–813. doi: 10.1089/10430340252898984

Funke, S., Maisner, A., Mühlebach, M. D., Koehl, U., Grez, M., Cattaneo, R., et al. (2008). Targeted cell entry of lentiviral vectors. *Mol Ther* 16, 1427–1436. doi: 10.1038/mt.2008.128

Hartmann, J., Münch, R. C., Freiling, R.-T., Schneider, I. C., Dreier, B., Samukange, W., et al. (2018). A Library-Based Screening Strategy for the Identification of DARPins as Ligands for Receptor-Targeted AAV and Lentiviral Vectors. *Mol Ther Methods Clin Dev* 10, 128–143. doi: 10.1016/j.omtm.2018.07.001

Jamali, A., Kapitza, L., Schaser, T., Johnston, I. C. D., Buchholz, C. J., and Hartmann, J. (2019). Highly Efficient and Selective CAR-Gene Transfer Using CD4- and CD8-Targeted Lentiviral Vectors. *Mol Ther Methods Clin Dev* 13, 371–379. doi: 10.1016/j.omtm.2019.03.003

Salmon, P., and Trono, D. (2007). Production and titration of lentiviral vectors. *Curr Protoc Hum Genet* Chapter 12, Unit 12.10. doi: 10.1002/0471142905.hg1210s54

Zufferey, R., Nagy, D., Mandel, R. J., Naldini, L., and Trono, D. (1997). Multiply attenuated lentiviral vector achieves efficient gene delivery in vivo. *Nat Biotechnol* 15, 871–875. doi: 10.1038/nbt0997-871
